# Detecting and distinguishing tipping points using spectral early warning signals

**DOI:** 10.1098/rsif.2020.0482

**Published:** 2020-09-30

**Authors:** T. M. Bury, C. T. Bauch, M. Anand

**Affiliations:** 1 Department of Applied Mathematics, University of Waterloo, Waterloo, Ontario, Canada ON N2L 3G1; 2 School of Environmental Sciences, University of Guelph, Guelph, Ontario, Canada ON N1G 2W1

**Keywords:** critical transition, early warning signal, population dynamics, power spectrum, bifurcation

## Abstract

Theory and observation tell us that many complex systems exhibit tipping points—thresholds involving an abrupt and irreversible transition to a contrasting dynamical regime. Such events are commonly referred to as critical transitions. Current research seeks to develop early warning signals (EWS) of critical transitions that could help prevent undesirable events such as ecosystem collapse. However, conventional EWS do not indicate the type of transition, since they are based on the generic phenomena of critical slowing down. For instance, they may fail to distinguish the onset of oscillations (e.g. Hopf bifurcation) from a transition to a distant attractor (e.g. Fold bifurcation). Moreover, conventional EWS are less reliable in systems with density-dependent noise. Other EWS based on the power spectrum (spectral EWS) have been proposed, but they rely upon spectral reddening, which does not occur prior to critical transitions with an oscillatory component. Here, we use Ornstein–Uhlenbeck theory to derive analytic approximations for EWS prior to each type of local bifurcation, thereby creating new spectral EWS that provide greater sensitivity to transition proximity; higher robustness to density-dependent noise and bifurcation type; and clues to the type of approaching transition. We demonstrate the advantage of applying these spectral EWS in concert with conventional EWS using a population model, and show that they provide a characteristic signal prior to two different Hopf bifurcations in data from a predator–prey chemostat experiment. The ability to better infer and differentiate the nature of upcoming transitions in complex systems will help humanity manage critical transitions in the Anthropocene Era.

## 1. Introduction

The understanding that complex systems can possess thresholds marking a sudden shift to an alternative dynamical regime has been around for a long time (e.g. in ecology [1,2]). Such a threshold may be referred to directly as a tipping point/catastrophic bifurcation, or by its inferred dynamics, a critical transition/regime shift. Predicting tipping points and their ensuing dynamics remains a significant challenge, since the observable state of a system may show little change right up until it is too late. Even where data are abundant, parametrized models based on biological principles are rarely able to pinpoint tipping points due to uncertainty in system parameters and mechanisms. However, a new wave of research is targeting stochasticity as a possible treasure trove of information on the otherwise hidden, and often surprising, dynamics of complex systems [3,4].

A significant development in this area is that of early warning signals (EWS), which are a suite of statistical metrics that are expected to undergo observable change prior to a tipping point [5,6]. Most EWS are grounded in the phenomenon of ‘critical slowing down’, which is a generic feature of local bifurcations [7]. It involves the degradation of restoring forces along some dimension of the system’s state space, resulting in a longer return time to equilibrium following a perturbation. In stochastic systems, this manifests as an increase in variance [8], higher correlations in time [9] and space [10], lower frequencies in the power spectrum [11], and notable changes in several other statistical metrics [4]. We use the term ‘conventional EWS’ to refer to these EWS that serve as proxies for critical slowing down.

The generality of critical slowing down is both a blessing and a curse. On one hand, it allows EWS to be applied to a wide range of systems including socio-ecological [12,13], neurological [14], financial [15] and climate [16] systems. On the other hand, bifurcations come in assorted forms which each possess their own unique dynamics [17], such as a smooth transition to an intersecting state, the onset of oscillations, or an abrupt departure to a distant attractor (e.g. Transcritical, Hopf, Fold bifurcations, respectively). These local bifurcations are all accompanied by critical slowing down [7] and so cannot be distinguished by the conventional critical slowing down methodology [18]. As such, EWS that are specific to each type of bifurcation are required to predict ensuing dynamics. Moreover, external noise that is correlated or density dependent can distort EWS [19,20], so there is a need to develop metrics that are more robust to these forms of noise.

The power spectrum has shown potential as a tool for detecting bifurcations from time-series data [11,21–24] and has a rich history in time-series analysis more generally [25,26]. A seminal study in the physics literature derived analytical approximations for its behaviour prior to bifurcations of periodic orbits [21], showing how different patterns emerge depending on the type of bifurcation. Later studies investigated how changes in the power spectrum could warn of an upcoming Fold bifurcation [11], and constructed EWS to pick up changes in spectral properties such as a spectral ratio [22] and the spectral exponent [23]. We refer to EWS based on the power spectrum collectively as ‘spectral EWS’. Current spectral EWS are based on the phenomenon of spectral reddening—the movement towards lower frequencies in the power spectrum as a bifurcation approaches—and have shown promise in providing warning of upcoming Fold bifurcations. However, if the bifurcation has an underlying oscillatory component (e.g. Hopf/Flip/Neimark–Sacker), which we argue is more likely in high-dimensional systems than simplified models would suggest (electronic supplementary material, Note), then spectral reddening does not occur, and these spectral EWS cannot be expected to provide a signal. Given that the power spectrum is a complete representation of a (stationary) time series, it possesses a lot of information. The question then becomes: how does one harness this information to provide maximum information about the underlying system?

For assessing and developing EWS, the theory of stochastic processes has much to offer [3,27–29], though most studies do not go beyond the rule of thumb provided by critical slowing down. This is well illustrated by the general framework of a system of variables ***s*** that evolves in time according to

1.1 ds=f(s) dt+σdW(t),

where *f* captures the within-system dynamics and d***W***(*t*) is a vector of Wiener processes representing environmental noise with amplitudes and correlations given in the matrix *σ*. For relatively small noise, the dynamics about an equilibrium state are well approximated by

1.2 dx=Ax dt+σ dW(t),

where ***x*** is the deviation of the state from equilibrium and *A* is the Jacobian matrix of *f* at equilibrium, which contains the local interaction terms between the variables. This process, originally studied in physics to model Brownian motion [30], is an Ornstein–Uhlenbeck process, for which general statistical properties can be derived [19,27]. Therefore, given a system that fits into this framework, analytical approximations for EWS can be derived in terms of system parameters (within *A*) and relative noise strengths and correlations (within *σ*). This way, one can move beyond generic indicators of an upcoming transition (which do not always behave as expected), towards more reliable indicators that are specific to the system being modelled.

This analytical approach was recently adopted in previous studies to investigate the behaviour of EWS specific to particular models [31,32], and different regimes of noise [20,28]. It has also been used to derive EWS approximations for a subset of local bifurcations in continuous-time [28] and analytical approximations of the power spectrum prior to continuous-time bifurcations of period orbits [21]. However, a more complete description of EWS in continuous-time is required to understand their behaviour prior to oscillatory bifurcations. This description should also include discrete-time systems, since a corresponding discrete-time model can exhibit fundamentally different dynamics (e.g. the logistic model for population growth exhibits steady-state dynamics in continuous-time, but regimes of oscillatory and chaotic dynamics in discrete-time [33]).

Here, we build on previous analytical work to derive EWS approximations for every local codimension-1 bifurcation in discrete and continuous-time systems (table 1). This provides a more complete framework for which EWS to expect preceding each type of bifurcation. We then develop two spectral EWS that are motivated by the insights from the analytical approximations. The first metric scales with the distance to the bifurcation in a favourable manner compared to conventional EWS, therefore providing a signal that is more likely to be detected. It is also more robust to density-dependent noise, and works for both oscillatory and non-oscillatory bifurcations, to which current spectral EWS are not suited. The second metric determines the class of bifurcations to which the upcoming instability belongs, allowing one to distinguish between certain types of transition. Finally, we apply the spectral EWS to model and empirical data, to demonstrate their ability to provide characteristic signals of different bifurcations.

**Table 1.** Analytical approximations for EWS preceding each local, codimension-1 bifurcation. Approximations are for the normal form of each bifurcation [17] with additive white noise of amplitude *σ*. The asymptotic behaviour of the peak in the power spectrum is most easily seen for the Fold bifurcation in continuous-time, where setting *ω* = 0 shows *S*_max_ scales like 1/*λ*^2^, whereas variance scales at the slower pace of 1/*λ*. Derivations use standard techniques from stochastic process theory [27], making the assumption of small noise (such that nonlinear terms are negligible) and quasi-stationarity (the bifurcation parameter varies sufficiently slowly). The dominant eigenvalue(s) is that of the Jacobian matrix about equilibrium. Shorthand notation includes TC, Transcritical; PF, Pitchfork, NS, Neimark–Sacker. Note that PF, Hopf, Flip and NS can be both super- and sub-critical. Expressions for the Fold, TC and PF in continuous-time are reported in [28]. Expressions for the power spectrum in continuous-time are reported in [21]. Derivations of expressions and asymptotic properties are provided in electronic supplementary material, Methods.

	bifurcation	dominant eigenvalue(s)	variance	lag-*τ* AC, *ρ*(*τ*)	power spectrum, *S*(*ω*)
continuous-time t,τ∈R	Fold, TC, PF	$\lambda \in R,\;\lambda \to 0^{-}$	$-\frac{\sigma^{2}}{2\lambda }$	e^*λ*|*τ*|^	$\frac{\sigma^{2}}{2\pi }(\frac{1}{\omega^{2}+\lambda^{2}})$
	Hopf	*λ*_1,2_ = *μ* ± *iω*_0_, *μ* → 0^−^	$-\frac{\sigma_{1}^{2}}{2\mu }$	e^*μ*|*τ*|^cos*ω*_0_*τ*	$\frac{\sigma_{1}^{2}}{4\pi }(\frac{1}{{\left(\omega -\omega_{0}\right)}^{2}+\mu^{2}}+\frac{1}{{\left(\omega +\omega_{0}\right)}^{2}+\mu^{2}})$
discrete-time t,τ∈Z	Fold, TC, PF	$\lambda \in R,\;\lambda \to 1^{-}$	$\frac{\sigma^{2}}{1-\lambda^{2}}$	*λ*^|*τ*|^	$\frac{\sigma^{2}}{2\pi }(\frac{1}{1+\lambda^{2}-2\lambda \cos(\omega )})$
	Flip	$\lambda \in R,\;\lambda \to -1^{+}$	$\frac{\sigma^{2}}{1-\lambda^{2}}$	*λ*^|*τ*|^	$\frac{\sigma^{2}}{2\pi }(\frac{1}{1+\lambda^{2}-2\lambda \cos(\omega )})$
	Neimark–Sacker	*λ*_1,2_ = *r e*^±*iθ*^, *r* → 1^−^	$\frac{\sigma_{1}^{2}}{1-r^{2}}$	*r*^|*τ*|^cos (*θτ*)	$\frac{\sigma_{1}^{2}}{4\pi }(\frac{1}{1+r^{2}-2r\cos(\omega -\theta )}+\frac{1}{1+r^{2}-2r\cos(\omega +\theta )})$

## 2. Results

### 2.1. Insights from analytical approximations

**Figure 1.**
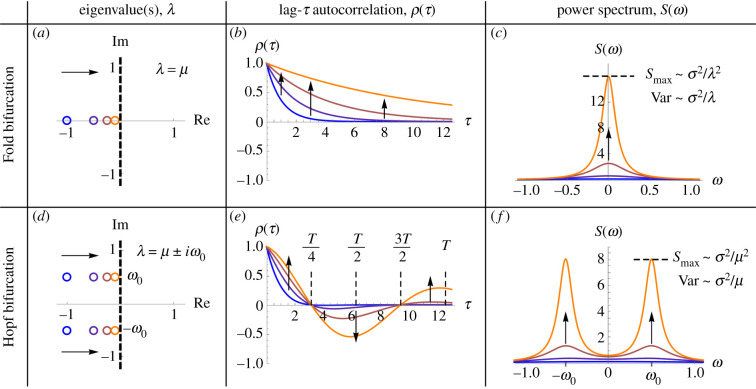
Contrasting analytical EWS preceding the Fold and the Hopf bifurcation. Analytical approximations for the autocorrelation function and power spectrum (table 1) are plotted at various distances to the bifurcation given by *μ* = { − 1, − 0.5, − 0.25, − 0.1}, where *μ* is the real part of the system’s dominant eigenvalue. (*a*) The Fold bifurcation involves a single real eigenvalue becoming positive. (*b*) Autocorrelation increases at all lag times, though very small lag times give a less noticeable increase, and large lag times yield a delayed increase. (*c*) The power spectrum becomes dominated by lower frequencies, with a peak amplitude that increases asymptotically faster than the variance (as *μ* → 0). (*d*) A Hopf bifurcation involves a complex-conjugate pair of eigenvalues obtaining positive real part. The imaginary part of the eigenvalues (*ω*_0_) corresponds to the frequency of oscillations that occur at the bifurcation. (*e*) The trend of autocorrelation depends on how the lag time compares with the underlying period of oscillations (*T* = 2*π*/*ω*_0_). For lag times near to half the period of oscillations, one in fact observes decreasing autocorrelation. (*f*) The power spectrum becomes dominated by *ω*_0_, with the peak amplitude increasing favourably compared to variance. Parameter values are *ω*_0_ = 0.5, *σ* = 1. Similar figures for each type of local codimension-1 bifurcation are provided in electronic supplementary material, figure S1.

The mathematical forms provided in table 1 reveal characteristic features of EWS that can be used to distinguish certain types of bifurcation. For example, the Fold and Hopf bifurcations are preceded by very different autocorrelation and power spectra (figure 1). The behaviour of these EWS preceding the other local codimension-1 bifurcations is shown graphically in electronic supplementary material, figure S1.

Consider lag-*τ* autocorrelation, which computes the correlation between data points spaced *τ* time units apart. The mathematical forms imply that bifurcations without an oscillatory component (Fold/Transcritical/Pitchfork) yield increasing autocorrelation for all lag times, conforming to the expected behaviour of critical slowing down (figure 1*b*). Choosing the most suitable lag time is not trivial however: smaller lag times can diminish the signal since nearby points are highly correlated even far from the bifurcation, and larger lag times can provide a delayed signal. By contrast, bifurcations with an oscillatory component (Hopf/Flip/Neimark–Sacker) yield an increasing *or* decreasing trend, that depends on the relationship between the lag time *τ*, and the underlying period of oscillations *T* (figure 1*e*). At sufficiently low lag times (*τ* < *T*/4), autocorrelation increases as the bifurcation is approached. However, for lag times near to half the period of oscillations, the autocorrelation decreases. This can be understood intuitively by noting that at a lag time of *T*/2, one is computing the correlation between peaks and troughs of the underlying frequency, which become more pronounced as the bifurcation is approached. Since these points occur on opposite sides of the trajectory mean, they possess negative correlation. This finding corroborates previous studies that have found decreasing autocorrelation in both empirical [34] and model [35] studies preceding oscillatory bifurcations. Studies that have found increasing autocorrelation prior to Hopf bifurcations [18,36] have used lag times much smaller than the period of oscillations. The analytical approximation for autocorrelation explains these previously contradictory outcomes. It also suggests that autocorrelation should be computed at multiple lag times, not just at lag-1, which is common practice in EWS studies.

The power spectrum provides perhaps the most intuitive picture of the approaching bifurcation. Prior to non-oscillatory bifurcations it moves to lower frequencies, which is a manifestation of critical slowing down, and coined ‘spectral reddening’ [11]. Prior to oscillatory bifurcations it moves to the frequency of oscillations that occur at the bifurcation. The fact that this frequency is observed in the power spectrum prior to the bifurcation is due to resonant amplification of stochasticity [37–39], which occurs in systems possessing damped oscillations subject to environmental or demographic noise.

### 2.2. Spectral early warning signals

We use the analytical expressions for the power spectrum to construct metrics (spectral EWS) that capture the important features that relate to the bifurcation. First, we use the peak in the power spectrum (*S*_max_) as an indicator of bifurcation proximity. This metric increases asymptotically like *σ*^2^/*μ*^2^, where *σ* is the external noise amplitude, and *μ* is the distance to the bifurcation. By contrast, variance increases like *σ*^2^/*μ*, which is a slower rate of increase as the bifurcation is approached (halving the distance to the bifurcation, doubles the variance, whereas *S*_max_ increases fourfold). Moreover, this scaling allows the metric to be more robust to changes in *σ* that may occur in systems with multiplicative or time-varying external noise. Second, we use AIC weights [40]—a metric that determines the relative parsimony of a set of models fitted to a dataset—to determine which bifurcation the measured power spectrum corresponds to. The ‘models’ here are the analytical forms for the power spectra preceding each bifurcation, and a flat power spectrum to serve as a null model (white noise). These spectral EWS should be used together as they provide complementary information about an upcoming transition. *S*_max_ warns of an upcoming bifurcation, and the AIC weights provide information on the type of transition to expect by indicating which analytical form most parsimoniously fits the power spectra.

### 2.3. Application to a model system

We test the spectral EWS on the classical Ricker model of a logistically growing population subject to harvesting (Methods). This model exhibits different bifurcations depending on the parameter that varies [35]. An increase in harvesting rate yields a Fold bifurcation to a diminished population state, whereas increasing the intrinsic growth rate of the population (which can occur through size-selective harvesting [41]) yields a Flip (period-doubling) bifurcation to an oscillatory regime. Whereas the Fold bifurcation results in a critical transition to an alternative state, the Flip bifurcation results in a smooth, and therefore reversible transition. Given these contrasting outcomes, it is important to be able to distinguish the upcoming bifurcation in advance. The EWS are computed on bootstrapped samples from segments of the time series within a rolling window (Methods), which provides the error bars in the EWS metrics. We emphasize that these error bars are not from multiple simulations of the system, but from the single trajectory as shown. We do this since, in reality, one usually only has a single realization to work with. To verify their trends over multiple simulations, we use Kendall tau values, which serve as a measure of increasing or decreasing trend. EWS from multiple realizations of this model are provided in electronic supplementary material, figures S2–S3.

**Figure 2.**
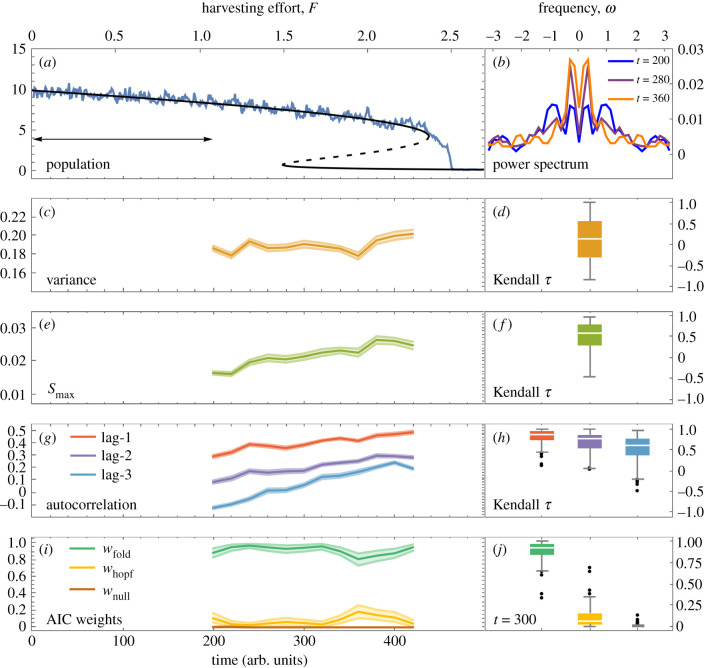
Characteristic EWS preceding the Fold bifurcation in the Ricker model. (*a*) Simulated response of population abundance (blue) to increasing harvesting effort superimposed on bifurcation diagram (black) of the deterministic model (solid, stable state; dashed, unstable state). Arrow illustrates the rolling window (40% of the time series) used for computing EWS. (*b*) Power spectrum becomes dominated by lower frequencies over time as expected for an approaching Fold bifurcation [11]. (*c*) Variance provides no useful signal due to density-dependent noise [19]. (*d*) Kendall tau values over 100 realizations show no consistent trend in variance. (*e*) *S*_max_ provides an increasing trend (*f* ) for the majority of realizations. (*g*) Autocorrelation provides an increasing trend, (*h*) with higher consistency at lower lag times. (*i*) AIC weights favour the Fold bifurcation. (*j*) At time *t* = 300, w_fold_ is dominant for 98 of 100 realizations. EWS are displayed as means with 95% confidence intervals over 100 bootstrapped samples from the time series in (*a*), after removing the trend with a Lowess filter. Kendall tau values are shown using box–whisker diagrams, where boxes mark the median and span the interquartile range and whiskers show the full range excluding outliers (black dots).

In the scenario undergoing the Fold bifurcation (figure 2),the trajectory shows a strong increasing trend in lag-1 autocorrelation, but no discernible trend in variance. The protocol of requiring increasing variance and lag-1 autocorrelation to signal a transition therefore fails in this case. This is an example of how an increase in variance does not necessarily precede transitions in systems with multiplicative noise [19]. However, since *S*_max_ is a more sensitive metric to changes in bifurcation proximity than variance, it still provides a signal prior to the bifurcation. The AIC weights correctly favour the power spectrum of the Fold bifurcation, and do so by the time *t* = 300 for 98 of the 100 realizations.

**Figure 3.**
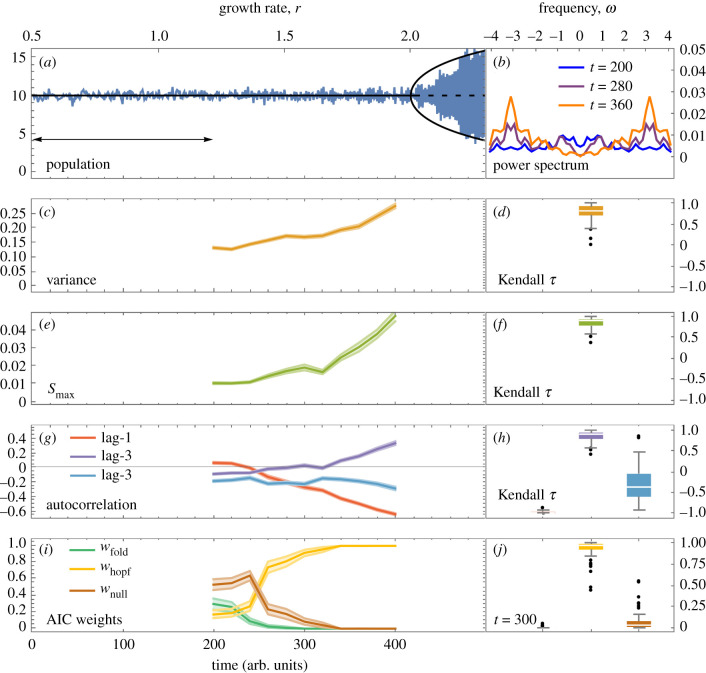
Characteristic EWS preceding the Flip bifurcation in the Ricker model. (*a*) Simulated response of population abundance (blue) to increasing growth rate superimposed on bifurcation diagram (black) of the deterministic model (solid, stable state/limit cycle; dashed, unstable state). Arrow illustrates the rolling window (40% of the time series) used for computing EWS. (*b*) Power spectrum becomes dominated by the frequency *ω* = *π*, in agreement with analytical approximations prior to a Flip bifurcation (table 1). (*c*) Variance provides an increasing trend, and (*d*) is confirmed to have an increasing trend over an ensemble of 100 realizations. (*e*) *S*_max_ yields an early warning signal with (*f*) a stronger increasing trend than variance. Note that while variance increases twofold, *S*_max_ increases fourfold, consistent with the asymptotic relationships of these metrics with the bifurcation parameter (figure 1). (*g*) Autocorrelation trend depends on the relationship between lag time and underlying period of oscillations (*T* = 2). Lag-1 autocorrelation decreases, since in this case the lag is half of the period (see figure 1). (*h*) Higher lags yield less pronounced signals on average. (*i*) AIC weights favour the power spectrum of a Hopf bifurcation, indicating upcoming oscillations. (*j*) At time *t* = 300, *w*_hopf_ dominates for 98 of 100 realizations. Computation details are as in figure 2.

The scenario undergoing the Flip bifurcation (figure 3) looks somewhat similar prior to the transition with regards to the population trend. The spectral EWS, however, tell a very different story, in agreement with the analytical approximations. Note that a Flip bifurcation from equilibrium yields oscillations of period *T* = 2 and so one expects an underlying frequency of *ω* = 2*π*/*T* = *π* prior to the transition, as observed in the power spectrum. Correspondingly, autocorrelation decreases at lag-1 and increases at lag-2 (seen analytically in figure 1*e*). *S*_max_ shows a marked increase (stronger than that of variance), and the AIC weights favour the power spectrum of a Hopf bifurcation, indicative of upcoming oscillations. The predicted frequency of these oscillations can be read off from the power spectrum as *ω*_0_ = *π*, in line with that of a Flip bifurcation. The spectral EWS together therefore provide a characteristic EWS of the Flip bifurcation that can be distinguished from the approaching Fold bifurcation.

**Figure 4.**
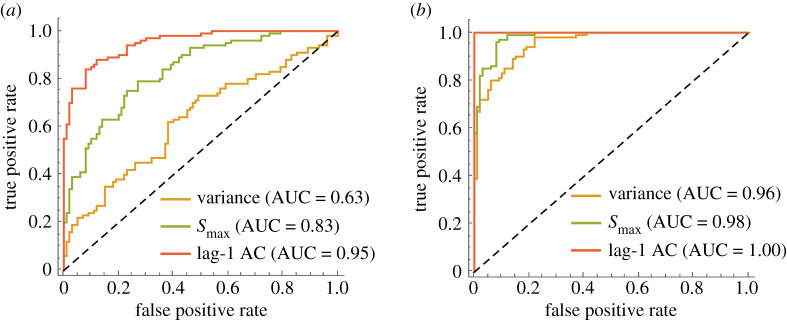
Receiver operator characteristics for EWS prior to the Fold (*a*) and Flip (*b*) bifurcation in the Ricker model. Area under the curve (AUC) is a measure of performance with values closer to one being better predictors of the transition. *Decreasing* lag-1 autocorrelation is used to predict the Flip bifurcation.

To assess the predictive power of the EWS, we compute receiver operator characteristics for forced and null simulations for each bifurcation (figure 4).We find that prior to both bifurcations, *S*_max_ outperforms variance as a warning signal (AUC = 0.83 versus 0.53 for the Fold bifurcation and AUC=0.98 versus 0.96 for the Flip bifurcation). Lag-1 autocorrelation provides the strongest prediction; however, it requires prior knowledge as to whether an increase or decrease is expected, which depends on the bifurcation. In this assessment, we take decreasing lag-1 autocorrelation as indication of the transition in the Flip scenario. To assess the efficacy of the EWS at alternative parameter values, we compute EWS over all combinations of the bifurcation parameters and find that the spectral EWS distinguish the transition in each case (electronic supplementary material, figures S4–S5).

### 2.4. Application to empirical data

**Figure 5.**
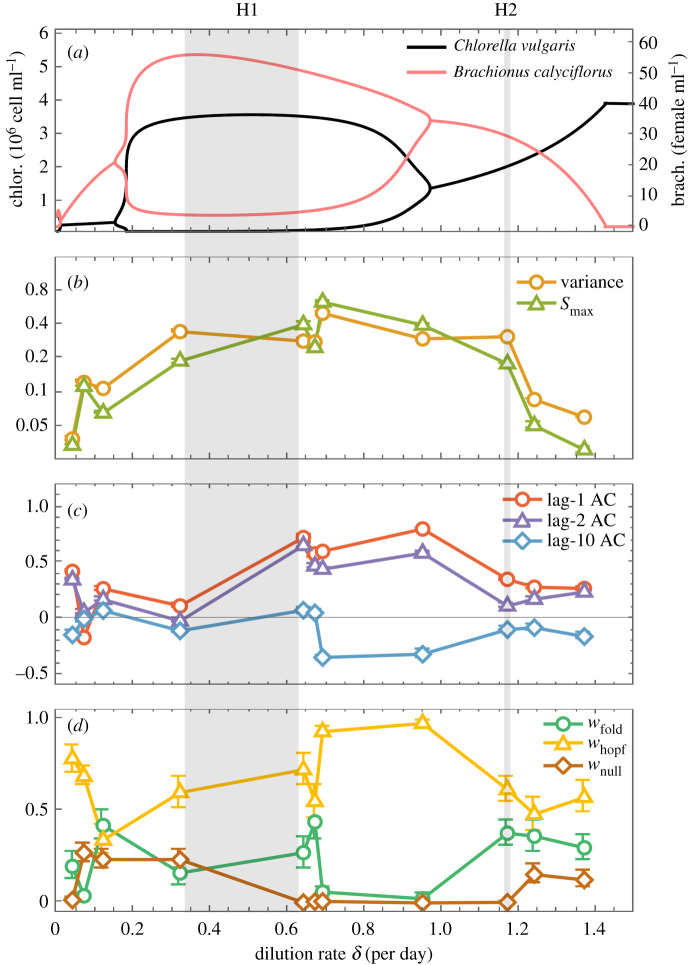
Characteristic early warning signals in an empirical predator–prey system. (*a*) Bifurcation diagram of a parametrized model [42] for the predator–prey dynamics between *Brachionus calyciflorus* and *Chlorella vulgaris*. Lines show stable states/limit cycles, indicating two Hopf bifurcations. Hopf bifurcations in the experiments (H1, H2) actually occur somewhere within the grey regions, which separate the oscillatory from equilibrium behaviour observed in the experiments [42]. (*b*) Variance and *S*_max_ as a function of dilution rate in experiments. Increasing trends are observed in *S*_max_ prior to the Hopf bifurcations. (*c*) Autocorrelation at low (1,2) and high (10) lag times provides no clear early warning signal. (*d*) The Hopf AIC weight dominates in the vicinity of the bifurcation, thus paired with *S*_max_, provides a characteristic early warning signal of an oscillatory bifurcation. Early warning signals are computed for 100 bootstrapped samples of each chemostat time series (fixed dilution rate), and displayed as means with 95% confidence intervals, across the samples and across the two species. Spectral EWS are also present in the time series of each species considered separately (electronic supplementary material, figure S7).

To test the spectral EWS in an empirical setting, we use chemostat data from a predator–prey experiment conducted in a previous study [42] (electronic supplementary material, figure S6). The authors showed using both experiments and a parametrized model that the system exhibits two Hopf bifurcations (H1, H2) as the dilution rate (controlling nutrient uptake) is varied (figure 5*a*). The observed Hopf bifurcations occur at slightly higher dilution rates than the model predicts [42], highlighting the difficulty of locating bifurcations in real systems, and the importance of having additional predictive tools such as EWS. We compute EWS for each experimental time series and find good agreement with theoretical expectations (figure 5*b*–*d*). Preceding both Hopf bifurcations, the spectral EWS provide a characteristic indication of a Hopf bifurcation—*S*_max_ shows an upward trend, and *w*_Hopf_ is the significant AIC weight. Unlike for H2, variance and *S*_max_ do fluctuate prior to H1, which is likely due to the data points being more tightly spaced in the region further from the bifurcation. In addition, the power spectrum in the pre-bifurcation regime of H1 (*δ* = 0.04) shows a dominant frequency, *ω*_0_ ≈ 1/3 (electronic supplementary material, figure S8) corresponding to an underlying period of oscillations, *T* = 2*π*(1/3)^−1^ ≈ 19 days, which approximates the period observed in the oscillatory regime. This suggests that in addition to determining the type of bifurcation, the power spectrum can provide an early estimate of the period of oscillations at the bifurcation.

## 3. Discussion

The ability to not only detect, but characterize bifurcations is essential for obtaining knowledge of upcoming qualitative changes in a system’s dynamics. In this paper, we have derived analytical expressions for EWS prior to each type of local bifurcation, enabling us to construct spectral EWS that distinguish oscillatory from non-oscillatory bifurcations and provide a more sensitive warning of changes in bifurcation proximity. We demonstrated these tools with a well-known population model, showing how the onset of collapse can be distinguished from the onset of oscillations, and applied them to empirical data of a population exhibiting a Hopf bifurcation. This paper shows that spectral EWS offer complementary information to conventional EWS and should be added to the repertoire of tools for predicting tipping points in real systems.

There exist other recent developments in tipping point detection that, under appropriate circumstances, show potential to predict specific bifurcations. Eigenvalue reconstruction [43] allows one to obtain an approximation of the Jacobian matrix from time-series data. This method permits the monitoring of stability loss via a diminishing eigenvalue, and the underlying frequency via the eigenvalue’s imaginary part (figure 1*a*,*d*). This approach, however, requires time-series data from multiple variables in order to make assertions on the type of bifurcation, whereas the power spectrum can be computed from an individual time series. Another approach considers the scaling of critical slowing down as a bifurcation is approached [14]; however, this requires a controlled setting whereby changes in the bifurcation parameter can be measured. Finally, promising studies in large perturbation theory [44,45] demonstrate how information on the bifurcation type and distance can be obtained by carefully monitoring a system’s recovery trajectory following a perturbation. This again requires a controlled environment, and a system whereby large perturbations are feasible.

This study uses ecological data from controlled chemostat experiments, where environmental conditions are kept fixed. Similar to other studies on EWS in experimental populations [46,47], this allows us to obtain estimates for the EWS at different values of the bifurcation parameter without requiring a rolling window. Our empirical results show that spectral EWS behave according to theoretical predictions in an empirical system. For real ecosystems however, environmental conditions vary over time, more similar to our model scenarios. Future studies should therefore test spectral EWS on high-resolution data of natural systems subject to time-varying environmental conditions. Unfortunately, many ecological datasets are too sparse for EWS to be effective [48]. However, technological advances in measurement devices are facilitating high-frequency data collection across many scientific disciplines. In ecology, sondes that are deployed on lakes to measure chlorophyll concentration are able to transmit data every minute, helping to anticipate algal blooms [49]. In medicine, wearable devices can now provide continuous physiological data to help detect disease transitions [50]. We anticipate that natural systems for which high-frequency data are available, will be those to most benefit from spectral EWS and EWS more generally.

Aside from detecting transitions, spectral EWS could contribute to a better understanding of systems where competing models exist. For example, the transition from quiescence to the spiking of neurons in the mammalian cortex has been modelled as both a Fold and Hopf bifurcation, depending on the underlying model assumptions [14]. Computing spectral EWS of empirical data, one could discern which bifurcation the transition corresponds to, providing stronger validation for one model over the other and therefore learning something about the underlying mechanisms of the system.

The findings of this study have to be seen in light of some limitations, which should be addressed in future studies. First, the spectral EWS proposed here only distinguish between oscillatory and non-oscillatory bifurcations, and not between bifurcations within these groups. For example, a supercritical and subcritical Hopf bifurcation, while giving rise to the same spectral EWS, result in structurally different transitions. Whereas the supercritical Hopf yields a smooth, reversible transition to small oscillations, the subcritical Hopf yields a critical, irreversible transition to a distant limit cycle. Distinguishing between such bifurcations requires consideration of nonlinear terms, which this study has assumed to be negligible—a valid assumption for systems subject to small noise relative to the width of the basin of attraction. Future studies should investigate how nonlinear terms alter the power spectrum prior to a bifurcation.

Second, this study only considers transitions arising due to local codimension-1 bifurcations. However, complex systems may lose stability via global bifurcations, which are not accompanied by critical slowing down, resulting in the failure of corresponding EWS [51,52]. In such situations, EWS based on other dynamical features are required, such as critical attractor growth, which has been shown to precede interior crises of excitable systems [53]. Given that the spectral EWS here are derived from changes in local dynamics, they cannot be expected to provide warning of global bifurcations. In addition, bifurcations of a higher codimension (number of parameters that must be varied for the bifurcation to occur), such as the Bautin bifurcation, remain little explored in the EWS literature yet arise in many models (e.g. [54]).

Third, spectral EWS, as with conventional EWS, may be distorted in cases of large noise, multiple scales of correlation and sparsely sampled data [48]. Further research should investigate the robustness of spectral EWS to these factors, and how the power spectrum varies in each case. For example, one would expect correlated environmental noise to create an additional peak in the power spectrum at the characteristic frequency of the noise. Finally, the work here does not consider spatial systems, where a power spectrum can be computed in both space and time. Investigating the behaviour of the power spectrum prior to different bifurcations in these systems may inspire further spectral EWS to predict and characterize transitions in spatial systems.

The spectral EWS developed here have several implications for science and policy. Competing hypotheses can lead to models with different types of bifurcations [14,55]. Monitoring time-series data with spectral EWS can be used to infer particular models based on their bifurcation structure, and therefore deepen our understanding of the system itself. To avoid a regime shift, action must be taken well in advance of the bifurcation [22], requiring indicators that respond quickly to changes in bifurcation proximity. Incorporating *S*_max_ may improve the likelihood of a sufficiently early warning due to its high sensitivity to bifurcation proximity. Moreover, the ability to distinguish oscillatory bifurcations (Hopf/Flip/Neimark–Sacker) from non-oscillatory ones is crucial, since their inferred dynamics produce contrasting outcomes. Our work here furthers methodology to learn useful information from stochasticity [3] and offers ready-to-go tools for further application.

## 4. Material and methods

### 4.1. Analytical derivations of early warning signals

Derivations use the normal form of each local bifurcation [17] with additive white noise. For example, derivations for the Fold bifurcation in continuous-time come from the system

4.1 $$\frac{\text{d}x}{\text{d}t}=\alpha -x^{2}+\sigma \xi (t),$$

where *α* is the bifurcation parameter, *ξ*(*t*) is a white noise process and *σ* is the noise amplitude. The corresponding normal form system in discrete-time is

4.2 $$x_{t+1}=x_{t}+\alpha -x_{t}^{2}+\sigma \epsilon_{t},$$

where now *ε*_*t*_ is a normal random variable of mean zero and unit variance. For each bifurcation, we linearize the system, yielding an Ornstein–Uhlenbeck process (in continuous-time) or a vector autoregression (in discrete-time). EWS for the corresponding stationary process are then derived from first principles using standard techniques from stochastic processes [25,27] (electronic supplementary material, Methods).

### 4.2. Population model

We use a Ricker-type model that describes the logistic growth of a population subject to harvesting [35]. The model reads

4.3 $$N_{t+1}=N_{t}{\text{e}}^{\left(r(1-N_{t}/K)+\sigma \epsilon_{t}\right)}-F\frac{N_{t}^{2}}{N_{t}^{2}+h^{2}},$$

where *N*_*t*_ is the population size at time *t*, *r* is the intrinsic growth rate, *K* is the carrying capacity, *F* is the maximum rate of harvesting, *h* is a half-saturation constant, *σ* is the noise amplitude and *ε*_*t*_ is a normal random variable with zero mean and unit variance. Baseline parameters are *r* = 0.75, *K* = 10, *F* = 0, *h* = 0.75, *σ* = 0.04. The model exhibits a Fold bifurcation at *F* = 2.36, and a Flip (period-doubling) bifurcation at *r* = 2.00 followed by a sequence of further Flip bifurcations to chaos.

We simulate two model scenarios, similar to a previous study [35]. In one, the harvesting rate *F* increases linearly over [0, 2.7], resulting in a Fold bifurcation. In the other, the growth rate *r* increases linearly over the interval [0.5, 2.3] resulting in a Flip bifurcation. All other parameters remain fixed. Both scenarios are simulated for 500 time-steps. Negative population values arising from noise are reset to zero. To test statistical significance of EWS, null trajectories are also simulated in which the bifurcation parameter is fixed.

### 4.3. Chemostat data analysis

Chemostat data were available for 14 different dilution rates [42] (electronic supplementary material, figure S8). The dilution rate was held constant in each of the experimental treatments and hence, as in the original study, the empirical Hopf bifurcations are considered to occur between two neighbouring dilution rates such that one dilution rate gives rise to observable oscillations and the other does not. For computing EWS, we considered only time series with greater than 25 data points, of which there were 11. The data were initially detrended with a Lowess filter with an 80 day span to account for any unintentional drift in the dilution rate.

### 4.4. Bootstrapping time-series data

Prior to computing EWS, we use block-bootstrapping to generate an ensemble of samples. This involves detrending the data using a Lowess filter, followed by using a rolling window to obtain overlapping segments that can be considered approximately stationary. For each segment, we generate 100 bootstrapped samples. Each sample is built by selecting blocks of the time series randomly with displacement. The block length is drawn from a geometric distribution [56], with an average large enough such that significant temporal correlations in the time series are retained.

### 4.5. Computing early warning signals

Conventional EWS were computed according to common practices [6]. The power spectrum was approximated using Welch’s method [57], which computes multiple periodograms from overlapping segments of the time-series data and averages them. The periodogram is given by $P(k)=|\tilde{x}(k){|}^{2}$ where

4.4 $$\tilde{x}(k)=\frac{1}{\sqrt{n}}\sum_{\;j=1}^{n}x_{j}{\text{e}}^{(2\pi i(j-1)(k-1))/n}$$

is the discrete Fourier transform, and *n* is the number of data points in each segment. From the resulting power spectrum *S*(*k*), we compute *S*_max_ = max_*k*_ *S*(*k*). The AIC weights *w*_fold_, *w*_hopf_ and *w*_null_ are found by fitting the canonical power spectrum forms

4.5 $$S_{\mathrm{fold}}(\omega ;\sigma ,\lambda )=\frac{\sigma^{2}}{2\pi }\frac{1}{\omega^{2}+\lambda^{2}},$$

4.6 $$S_{\mathrm{hopf}}(\omega ;\sigma ,\mu ,\omega_{0})=\frac{\sigma^{2}}{2\pi }\left(\frac{1}{{\left(\omega -\omega_{0}\right)}^{2}+\mu^{2}}+\frac{1}{{\left(\omega +\omega_{0}\right)}^{2}+\mu^{2}}\right)$$

4.7 $$\;\text{and}\;S_{\mathrm{null}}(\omega ;\sigma )=\frac{\sigma^{2}}{2\pi }\;$$

to the measured power spectrum using a nonlinear optimization procedure, and taking appropriate ratios of the AIC scores [40]. Finer details are provided in electronic supplementary material, Methods. In addition, we have developed *ewstools*, an open-source Python package located at https://github.com/ThomasMBury/ewstools and available on the Python Package Index. To date, *ewstools* provides general functionality for computing conventional and spectral EWS, including auxiliary methods such as bootstrapping. We intend to continue expanding the package and welcome contributions from other Python users.

## Supplementary Material

Supplementary figures, methods and notes

## Data accessibility

The chemostat data used in this study can be obtained from Gregor Fussmann upon reasonable request.
