# Supplementary material for: Detecting and distinguishing tipping points using spectral early warning signals
Source: J R Soc Interface. 2020 Sep 30;17(170):20200482. doi: 10.1098/rsif.2020.0482 (PMC7536046; doi:10.1098/rsif.2020.0482)
Supplement: Supplementary figures, methods and notes [file rsif20200482supp1.pdf]

Journal of the Royal Society Interface

Detecting and Distinguishing Tipping Points using Spectral Early Warning Signals: Supplementary Information

Thomas M. Bury[1,2*], Chris T. Bauch[1], Madhur Anand[2]

1 Dept. of Applied Mathematics, University of Waterloo
2 School of Environmental Sciences, University of Guelph

* tbury@uwaterloo.ca

Contents

1 Supplementary Figures **2**

2 Supplementary Methods **11**

2.1 Computation of EWS from time series data 11

2.2 Analytical approximations of EWS 16

2.3 Asymptotic properties of $S_{\max}$ 24

3 Supplementary Note **26**

3.1 Local bifurcations in higher-dimensional systems 26

Glossary **31**

1 Supplementary Figures

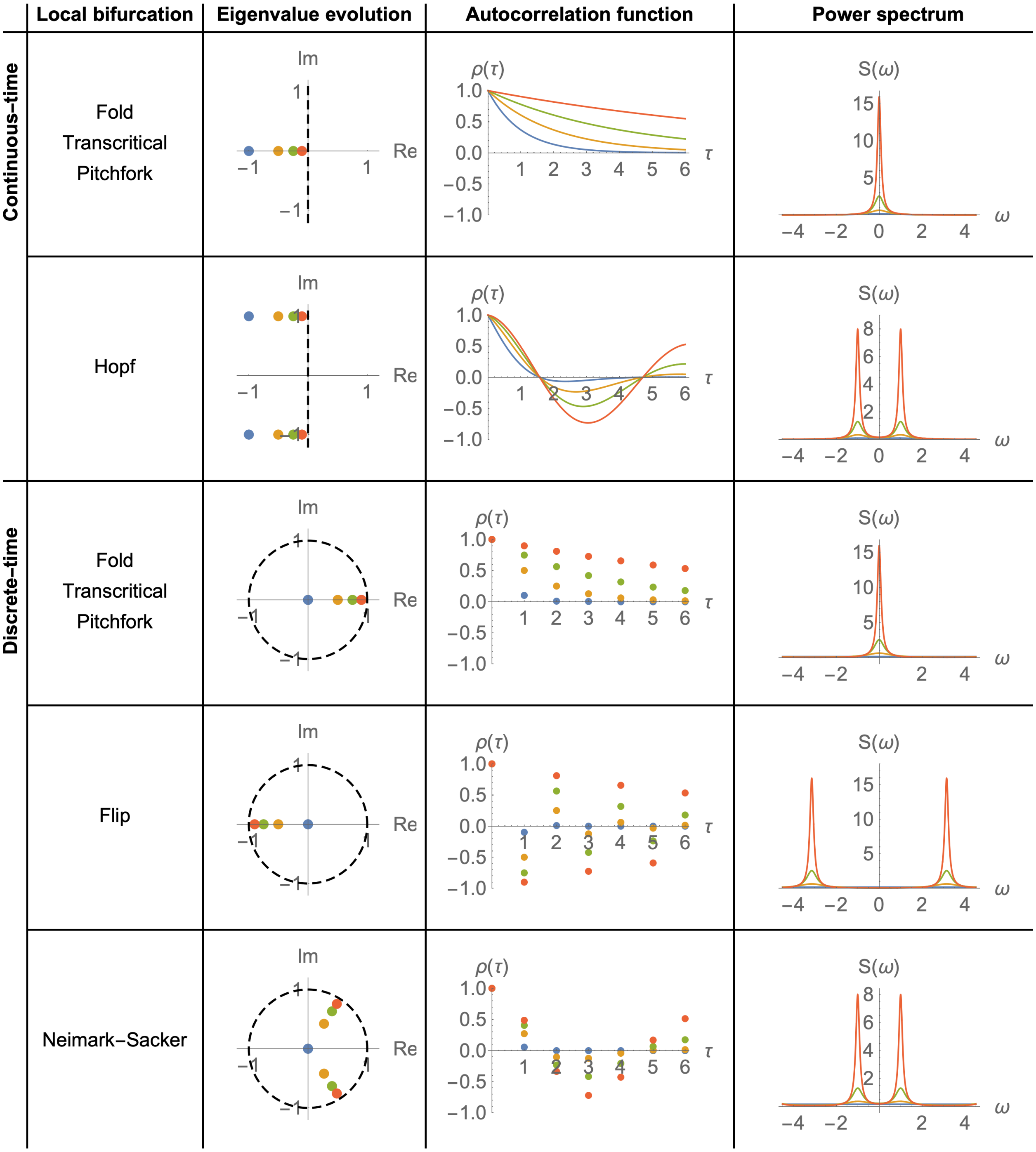

Supplementary Figure 1. Analytical approximations of EWS preceding each type of local codimension-one bifurcation. We show the analytical approximation of the autocorrelation function $\rho(\tau)$ and power spectrum $S(\omega)$ at different proximities to each bifurcation given by eigenvalues of the Jacobian matrix. For the continuous-time bifurcations, we show $\text{Re}(\lambda) = \{-1, -0.5, -0.25, -0.1\}$. For the discrete-time bifurcations we show $|\lambda| = \{0, 0.5, 0.75, 0.9\}$. Other parameters include the frequency at the Hopf/Neimark-Sacker bifurcation ($\omega_0 = 1$), and the noise amplitudes ($\sigma_1 = \sigma_2 = 1$).

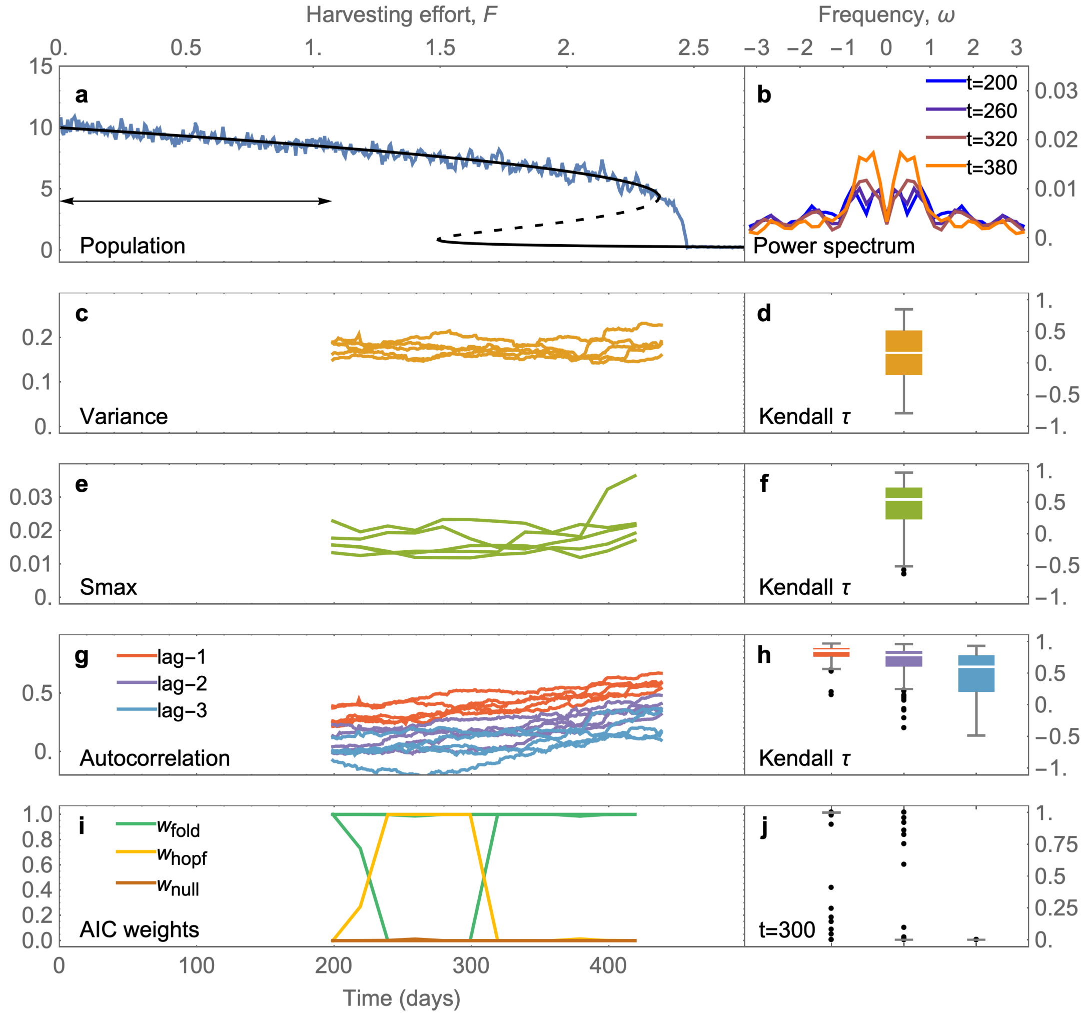

Supplementary Figure 2. EWS in the Ricker model: a Fold bifurcation **a,** Sample realisation of population density (blue) superimposed on bifurcation diagram. Solid (dashed) black lines represent stable (unstable) states. Arrow illustrates the rolling window (40% of the time series) used for computing EWS. **b,** Power spectrum of (a) within the rolling window at distinct times. **c,** Variance of 5 sample realisations. **d,** Kendall tau values of variance trends over 100 realisations. **e,** $S_{\max}$ of 5 sample realisations. **f,** Kendall tau values for $S_{\max}$ trends. **g,** Autocorrelation of 5 sample realisations at lags 1, 2 and 3. **h,** Kendall tau values of autocorrelation trends. **i,** AIC weights of 5 sample realisations. **j,** AIC weights at time $t = 300$, over 100 realisations. The model (Methods) is simulated over 500 time steps with linearly increasing F within the interval $[0, 2.7]$. Realisations are detrended with a Lowess filter of span 0.5 before EWS are computed. Kendall tau values are shown using box-whisker diagrams, where boxes mark the median and span the interquartile range and whiskers show the full range excluding outliers (black dots) from the ensemble of realisations.

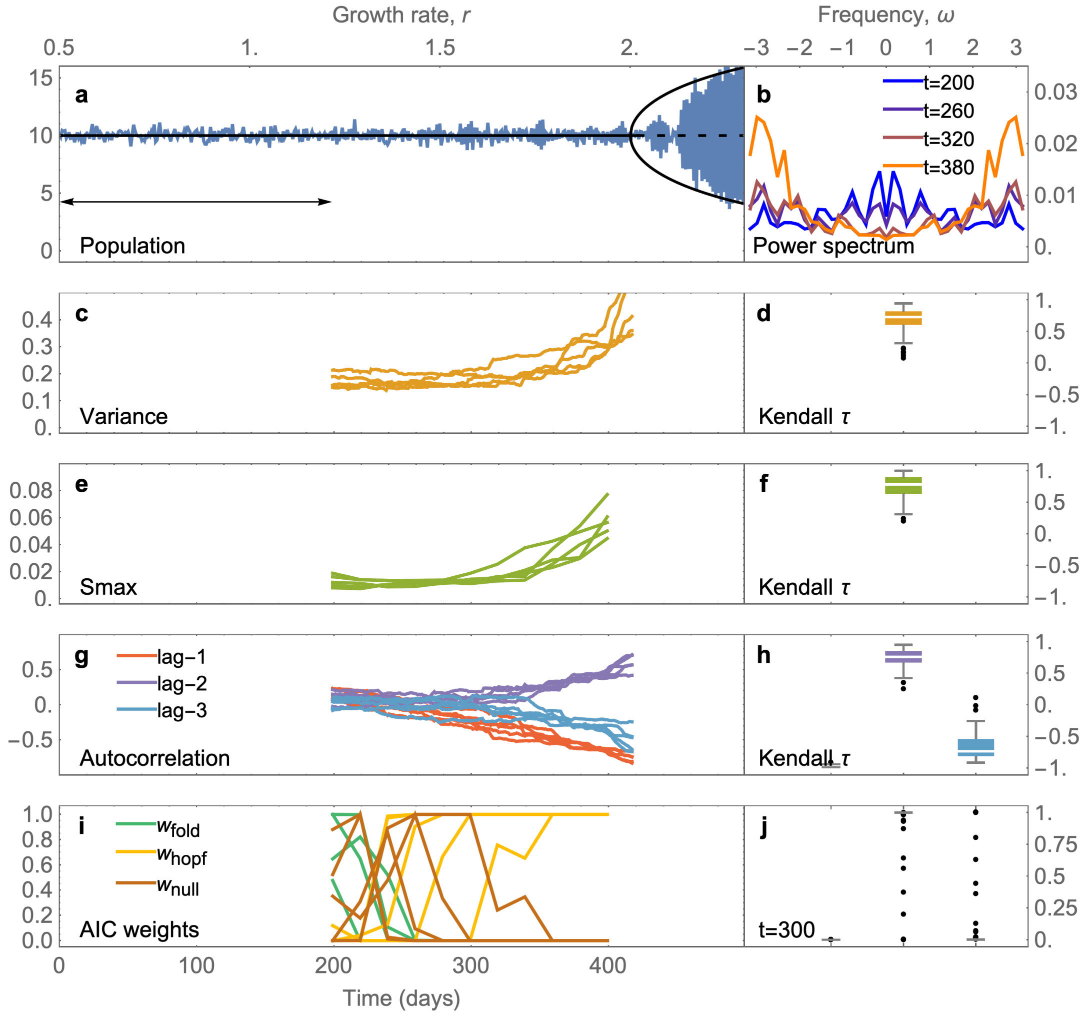

Supplementary Figure 3. EWS in the Ricker model: a Flip bifurcation a, Sample realisation of population density (blue) superimposed on bifurcation diagram. Solid (dashed) black lines represent stable (unstable) states/limit cycles. Arrow illustrates the rolling window (40% of the time series) used for computing EWS. **b,** Power spectrum of (a) within the rolling window at distinct times. **c,** Variance of 5 sample realisations. **d,** Kendall tau values of variance trends over 100 realisations. **e,** S_{max} of 5 sample realisations. **f,** Kendall tau values for S_{max} trends. **g,** Autocorrelation of 5 sample realisations at lags 1, 2 and 3. **h,** Kendall tau values of autocorrelation trends. **i,** AIC weights of 5 sample realisations. **j,** AIC weights at time $t = 300$, over 100 realisations. The model (Methods) is simulated over 500 time steps with linearly increasing r within the interval $[0.5, 2.3]$. Realisations are detrended with a Lowess filter of span 0.5 before EWS are computed. Kendall tau values are shown using box-whisker diagrams, where boxes mark the median and span the interquartile range and whiskers show the full range excluding outliers (black dots) from the ensemble of realisations.

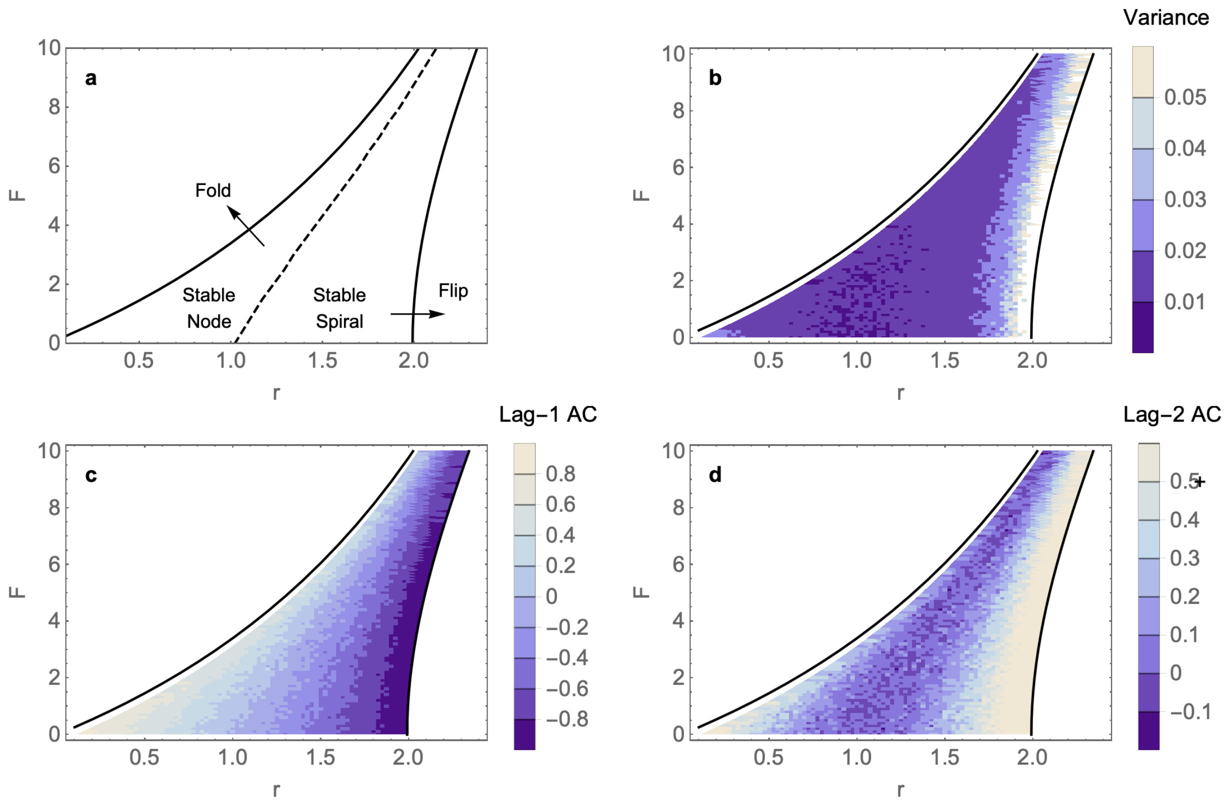

Supplementary Figure 4. Conventional EWS in the bifurcation parameter plane of the Ricker model. For fixed values of (r, F) within the stable region of the bifurcation plane, we simulate the Ricker model for 400 time steps and compute the conventional EWS. **a.** Regions of stability and bifurcation curves. Crossing the Flip (Fold) bifurcation curve results in a transition to oscillations (an alternate stable state). Perturbations in the stable node (stable spiral) regime result in a monotonic (oscillatory) recovery trajectory. **b.** Variance. **c.** Lag-1 autocorrelation. **d.** Lag-2 autocorrelation.

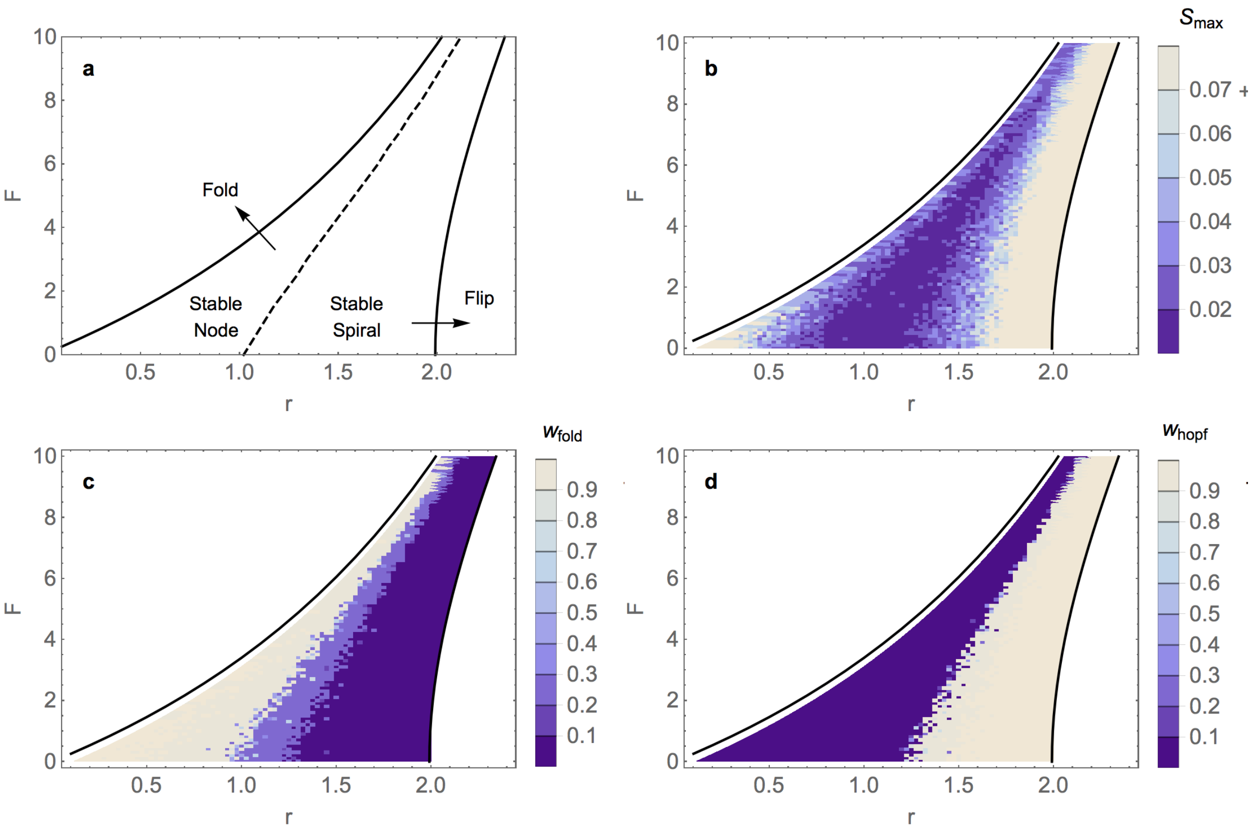

Supplementary Figure 5. Spectral EWS in the bifurcation parameter plane of the Ricker model. For fixed values of (r, F) within the stable region of the bifurcation plane, we simulate the Ricker model for 400 time steps and compute the spectral EWS. **a.** Regions of stability and bifurcation curves. Crossing the Flip (Fold) bifurcation curve results in a transition to oscillations (an alternate stable state). Perturbations in the stable node (stable spiral) regime result in a monotonic (oscillatory) recovery trajectory. **b.** Peak in the power spectrum $S_{\max}$. **c.** Fold AIC weight. **d.** Hopf AIC weight.

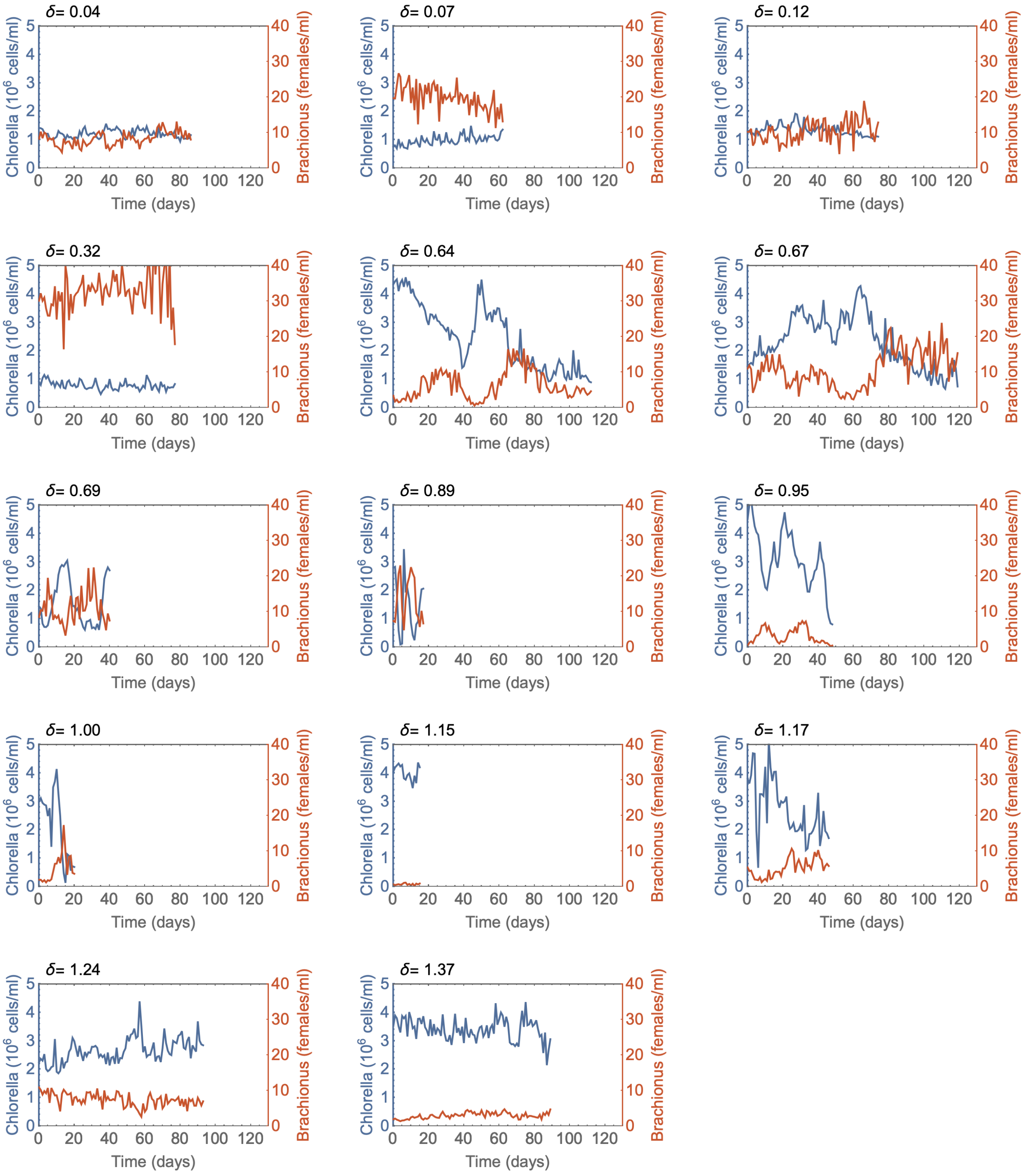

Supplementary Figure 6. Predator-prey trajectories from chemostat experiments (adapted from Fussmann et al[1]). Time-series data of the prey species (*Chlorella*, blue) and the predator species (*Brachionus*, red) from chemostat experiments run at different dilution rates (δ). The sampling frequency is one measurement per day. A Hopf bifurcation was conjectured to occur in the range $0.32 < \delta < 0.64$ and at $\delta \approx 1.16$, based on the system's transition from equilibrium to oscillatory behaviour either side of these thresholds.

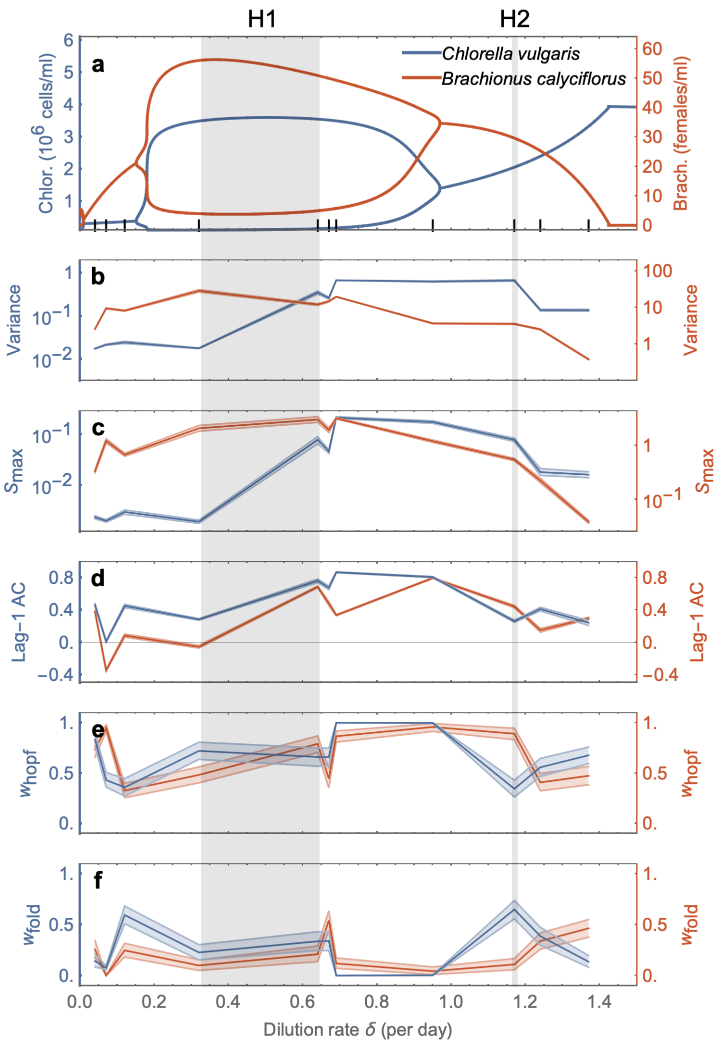

Supplementary Figure 7. Bootstrapped EWS by species in the empirical predator-prey system. For each chemostat time-series (with a fixed dilution rate δ), we computed 100 samples of bootstrapped EWS (Methods) and display the means with 95% confidence intervals. **a.** Bifurcation diagram of parametrised model[1] for Chlorella (blue) and Brachionus (red). Lines show stable states / limit cycle. Grey areas show regions where a Hopf bifurcation actually took place in experiments.[1] Bold ticks show the dilution rates used in experiments. **b.** Variance. **c.** Lag-1 autocorrelation. **d.** Peak in the power spectrum. **e-f.** AIC weights. Note that w_{null} can be obtained from the relation $w_{\text{fold}} + w_{\text{hopf}} + w_{\text{null}} = 1$. We computed EWS using a Hamming window of 80 days and a bootstrap block size of 40 days.

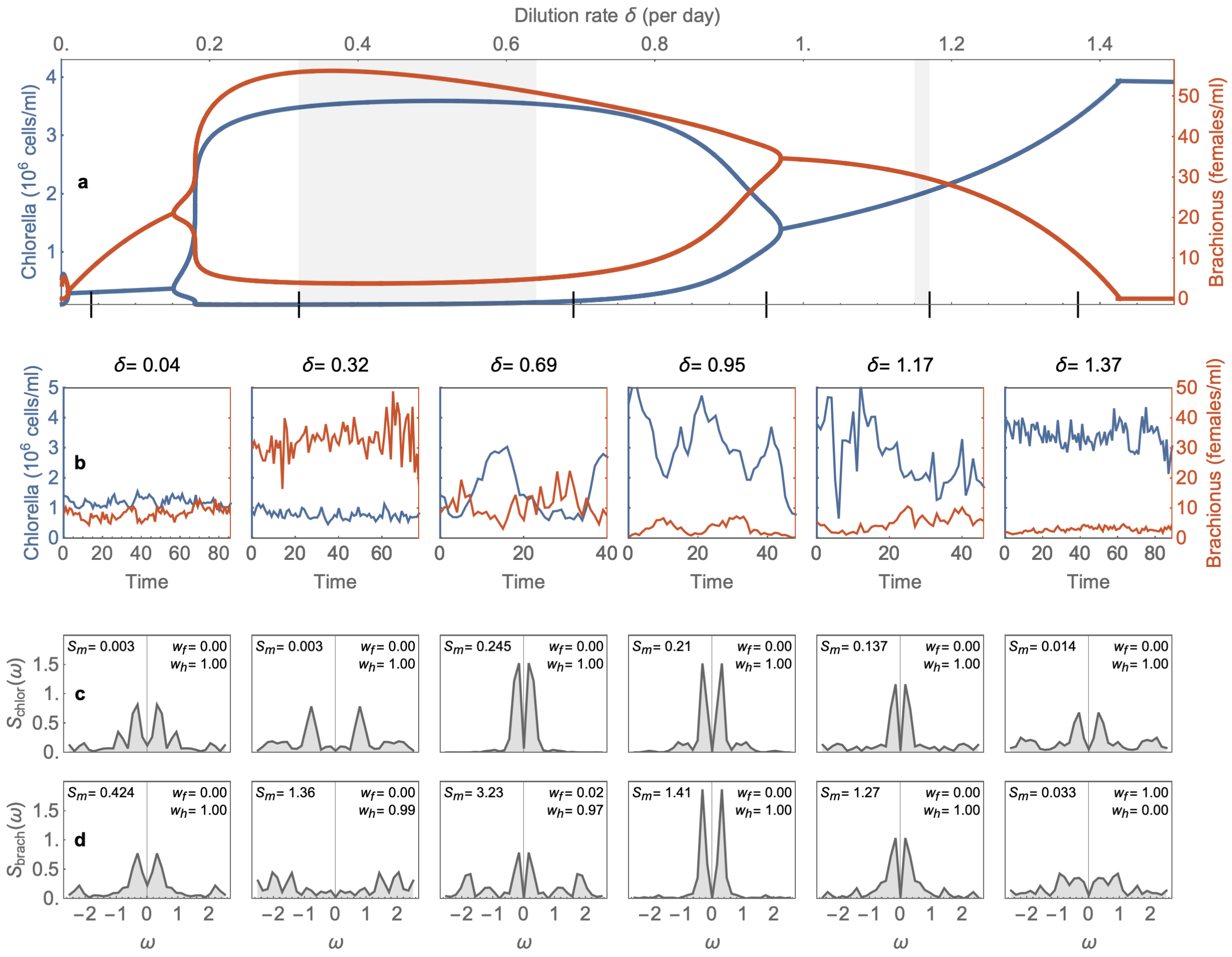

Supplementary Figure 8. Spectral EWS for the empirical predator-prey system. a. Bifurcation diagram of the model system showing equilibria / extreme points of limit cycles for the prey (*Chlorella*, blue) and predator (*Brachionus*, red). The two grey regions mark where the two Hopf bifurcations occur in the empirical system. Black dashes mark the dilution rates shown in b. **b.** Empirical time-series data from chemostats with the given dilution rate (δ). **c-d,** Normalised power spectra of the time-series data in b for *Chlorella* and *Brachionus* respectively. Data insets give the peak power (S_{max}), the Fold AIC weight (w_{f}) and the Hopf AIC weight (w_{h}).

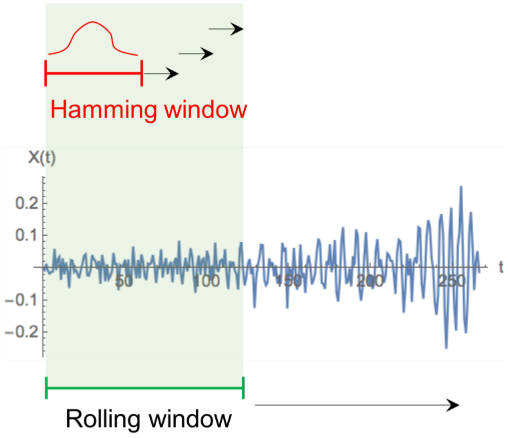

Supplementary Figure 9. Illustration of rolling window and Hamming window used to compute power spectrum. The rolling window moves incrementally across the residual time-series. At each increment, the power spectrum of the data within the window is estimated. This is done by computing the periodogram across several Hamming windows with a specified length and offset, and computing their average. This gives a more consistent, unbiased estimate of the power spectrum than a single periodogram using the full length of the rolling window.[3]

2 Supplementary Methods

2.1 Computation of EWS from time series data

This section provides further details on the methods used to compute temporal and spectral early warning signals of time-series data. These procedures may be simply implemented using the Python package *ewstools*, which is available and maintained at https://github.com/ThomasMBury/ewstools.

2.1.1 Bootstrapping time series data.

In some cases, we used block-bootstrapping to provide error bounds on the EWS calculations within a single time series. This involves detrending the data using a Lowess filter, followed by using a rolling window to obtain overlapping segments that can be considered approximately stationary. For each segment, we generate 100 bootstrapped samples. Each sample is built by selecting blocks of the time series randomly with displacement. The block length is drawn from a geometric distribution [2], with an average large enough such that significant temporal correlations in the time series are retained.

2.1.2 Temporal EWS

Temporal EWS, including variance and autocorrelation, are computed according to common practices.[4] We initially detrend the data using a Lowess function with a specified span. The EWS metrics are then computed over a rolling window of size chosen such that the system can be considered approximately stationary within that window. For data points within the window $\{x_j\}_{j=1}^n$, the variance is computed as

$$\text{Var} = \frac{1}{n}\sum_{j=1}^{n}(x_j - \mu)^2 \tag{1}$$

where $\mu = \sum_{j=1}^n x_j$, the mean value of the data. The lag-τ autocorrelation is computed as

$$\phi(\tau) = \frac{\frac{1}{n}\sum_{j=1}^{n-\tau}(x_j - \mu)(x_{j+\tau} - \mu)}{\text{Var}}. \tag{2}$$

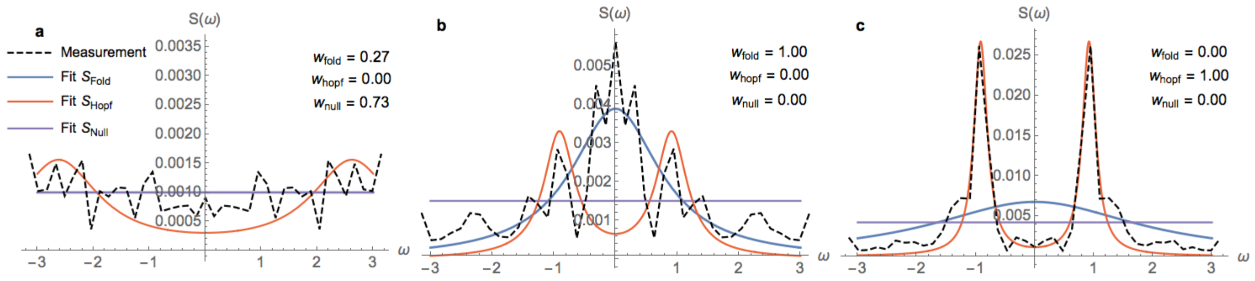

Supplementary Figure 10. Illustration of computing AIC weights in different dynamic regimes. For each power spectrum measurement, the analytical forms S_{fold}, S_{hopf} and S_{null} are fitted using nonlinear optimisation procedures. The AIC weights for each fit are then computed (goodness of fit, relative to the other models). **a.** Equilibrium system far from a bifurcation with strong white noise. **b.** Power spectrum in the vicinity of a Fold bifurcation. **c.** Power spectrum in the vicinity of a Hopf bifurcation.

2.1.3 Spectral EWS

To compute spectral EWS, we first need an approximation of the power spectrum, which we get using Welch's method [3, 5]. This approximates the power spectrum as the average of periodograms, computed over many overlapping Hamming windows, as illustrated in Figure 9. Data within the Hamming window $\{x_j\}_{j=1}^m$ is weighted by the function

$$W(j) = \frac{21}{46}\cos\left(\frac{2\pi j}{n} - \pi\right) + \frac{25}{46}, \qquad j \in [1, m], \tag{3}$$

to obtian the transformed data $\{y_j\}_{j=1}^m$. The periodogram is then computed as $P(k) = |\tilde{y}(k)|^2$, where

$$\tilde{y}(k) = \frac{1}{\sqrt{n}} \sum_{j=1}^{n} x_j e^{\frac{2\pi i (j-1)(k-1)}{n}}, \tag{4}$$

is the Discrete Fourier Transform of $\{y_j\}_{j=1}^m$. Averaging the obtained periodograms in this way gives a more consistent, unbiased estimate of the power spectrum than a single periodogram over the full length of the rolling window [3].

The peak in the power spectrum S_{max} is computed from the power spectrum $\{S(k)\}_{k=1}^n$ as

$$S_{\text{max}} = \max_k S(k). \tag{5}$$

The AIC weights [6] are computed from the fitting of the canonical power spectrum forms

$$S_{\text{null}}(\omega; , \sigma) = \frac{\sigma^2}{2\pi}, \tag{6}$$

$$S_{\text{fold}}(\omega; \sigma, \lambda) = \frac{\sigma^2}{2\pi}\frac{1}{\omega^2 + \lambda^2}, \tag{7}$$

$$S_{\text{flip}}(\omega; , \sigma, r) = \frac{\sigma^2}{2\pi}\frac{1}{1 + r^2 - 2r\cos\omega} \tag{8}$$

$$S_{\text{hopf}}(\omega; \sigma, \mu, \omega_0) = \frac{\sigma^2}{4\pi}\left(\frac{1}{(\omega - \omega_0)^2 + \mu^2} + \frac{1}{(\omega + \omega_0)^2 + \mu^2}\right). \tag{9}$$

to the measured power spectrum, as illustrated in Figure 10. S_{null} is the power spectrum for white noise. Systems far from a bifurcation under a strong white noise regime have a relatively flat spectrum, best captured by this fit.

The models are fit to the measured power spectrum using a nonlinear optimisation algorithm (Levenberg–Marquardt) from the open-source Python package LMFIT [7]. Further details on the nonlinear optimisation procedure are provided in the following section. The AIC scores are given by

$$\phi_i = -2\text{Log}L_i + 2V_i, \qquad i = 1 \dots n \tag{10}$$

where L_i is the maximum likelihood for the candidate model i, and V_i is the number of free parameters that model i possesses. The model with the lowest AIC score is the one which statistically best explains the data. Note that models are penalised for having extra parameters, so although S_{hopf} can fit a unimodal power spectrum just as well as S_{fold} (by setting $\omega_0 = 0$) it obtains a higher AIC score due to its extra parameter.

The AIC weights provide a normalised metric that discriminates between the models [8]. They are computed as follows. First, we obtain the difference between each score ϕ_i, and the score corresponding the model with the best fit [8]

$$\Delta_i = \phi_i - \text{min}_i \phi_i. \tag{11}$$

The AIC weights are then determined by

$$w_i = \frac{e^{-\frac{1}{2}\Delta_i}}{\sum_{i=1}^{n} e^{-\frac{1}{2}\Delta_i}}. \tag{12}$$

Note that the AIC weights are normalised and so sum up to 1. Values of a weight closer to 1 favour the corresponding model.

2.1.4 Nonlinear optimisation procedure

As with any computationally feasible optimisation algorithm, the procedure can output a local minimum without reaching the global minimum. Moreover, the local minimum that the algorithm reaches is dependent on the initial parameter guess that it is given. As such, we sweep over a range of initial parameter guesses and store the best model fit (smallest squared error).

Model constraints For the fitting procedure, we impose some logical constraints on the parameters. This avoids the optimisation algorithm searching through irrelevant parameter combinations, thereby reducing computational time. The constraints we impose are as follows:

$$\sigma > 0, \tag{13}$$
$$\lambda, \mu < 0, \tag{14}$$
$$-1 < r < 0, \tag{15}$$
$$S_{\text{fold}}(\omega_{\text{max}}) < \psi_f S_{\text{fold}}(0), \tag{16}$$
$$S_{\text{hopf}}(0) < \psi_h \, S_{\text{hopf}}(\omega_0). \tag{17}$$

Constraint (13) is imposed by the definition of the noise amplitude (a standard deviation of a distribution). We impose constraint (14), since we are only considering systems in a stable state (λ and μ correspond to the real part of the eigenvalues for the system's Jacobian matrix, which must be negative for the system to be stable). Constraint (15) restricts S_{flip} to a stable regime closer to a Flip bifurcation than a Fold bifurcation. Constraint (16) asserts that the power at the edge of the frequency domain must be less than some proportion $\psi_f \in [0, 1]$ of the power at the zero-frequency. Note that this is guaranteed sufficiently close to a Fold bifurcation since $S_{\text{fold}}(0) \to \infty$ as $\lambda \to 0$, whereas $S_{\text{fold}}(\omega_{\text{max}})$ is always finite. Our motivation for imposing this constraint is to better distinguish S_{fold} from the flat spectrum S_{null} which signifies no imminent bifurcation.

Constraint (17) asserts that the power at the zero-frequency is at most a proportion $\psi_h \in [0, 1]$ of the power at the intrinsic frequency of the Hopf bifurcation ω_0. This condition is always satisfied sufficiently close to a Hopf bifurcation since as $\mu \to 0$, $S_{\text{hopf}}(\omega_0) \to \infty$, whereas $S_{\text{hopf}}(0)$ always remains finite. Our motivation for this constraint is to distinguish S_{hopf} from S_{fold}, since without this constraint, S_{hopf} can fit a unimodal power spectrum just as well as S_{fold} can by choosing $\omega_0 = 0$, and a Hopf bifurcation may be erroneously flagged. With the constraint, this power spectrum models fit only the two distinct behaviours that are expected preceding the Fold and Hopf bifurcations respectively.

The constraints (16) and (17) are algebraically equivalent to the parameter constraints

$$\omega_0^2 > \frac{\mu^2}{4\psi_h}\left(4 - 3\psi_h + \sqrt{\psi_h^2 - 16\psi_h + 16}\right), \tag{18}$$

$$\lambda^2 < \left(\frac{\psi_f}{1 - \psi_f}\right)\omega_{\text{max}}^2, \tag{19}$$

respectively, which are coded into the optimisation algorithm. We take the values $\psi_f = 0.2$, $\psi_h = 0.5$.

Initial parameter guesses The analytical forms for the power spectra are fitted to the measured power spectrum using nonlinear optimisation procedures from the open-source Python package LMFIT [7]. Specifically, the Levenberg–Marquardt algorithm is used to minimise the least square error of the fit. As with any computationally feasible optimisation algorithm, the procedure can output a local minimum without reaching the global minimum. Moreover, the local minimum that the algorithm reaches is dependent on the initial parameter guess that it is given. As such, we sweep over a range of initial parameter guesses and store the best model fit (smallest squared error).

Appropriate initial parameter guesses are made based on three characteristic metrics of the power spectrum values $\{(\omega_i, S(\omega_i))\}_i$. This allows the algorithm to generalise to power spectra on significantly different scales. (For parameter guesses that are independent of the power spectrum, this would have been a problem). Consider the following metrics

$$S_{\text{max}} = \max_i S(\omega_i) \tag{20}$$

$$\omega_{\text{dom}} = \arg\max_i S(\omega_i) \tag{21}$$

$$S_{\text{tot}} = \sum_i S(\omega_i)\Delta\omega \tag{22}$$

where $\Delta\omega$ is the difference between consecutive frequency values. Note that each of these metrics can be computed from the measured spectrum with very little computational effort. Now, we can find the equivalent metrics using the analytical forms to provide a functional relationship between the metrics and the parameter values. Then, given numerical values for these metrics, we can compute the corresponding parameter values, and they will serve as our scale-independent initial parameter guesses. The mathematical forms for these initial parameter guesses are derived below.

For fitting S_{fold}, our baseline initial parameters $(\tilde{\sigma}, \tilde{\lambda})$ are set to satisfy

$$S_{\text{max}} = \frac{\tilde{\sigma}^2}{2\pi\tilde{\lambda}^2}, \qquad S_{\text{tot}} = -\frac{\tilde{\sigma}^2}{2\tilde{\lambda}} \tag{23}$$

which are the analytical expressions for the peak in $S_{\text{fold}}(\omega)$ and its total variation respectively. This gives an initialisation of

$$\tilde{\sigma} = \sqrt{\frac{2}{\pi}}\frac{S_{\text{tot}}}{\sqrt{S_{\text{max}}}}, \qquad \tilde{\lambda} = -\frac{S_{\text{tot}}}{\pi S_{\text{max}}}. \tag{24}$$

For fitting S_{flip}, our baseline initial parameters $(\tilde{\sigma}, \tilde{r})$ are set to satisfy

$$S_{\max} = \frac{\tilde{\sigma}^2}{2\pi(1+\tilde{r})^2}, \qquad S_{\text{tot}} = \frac{\tilde{\sigma}^2}{1-\tilde{r}^2} \tag{25}$$

which gives an initialisation of

$$\tilde{r} = -\frac{2\pi S_{\max} - S_{\text{tot}}}{2\pi S_{\max} + S_{\text{tot}}}, \quad \tilde{\sigma} = \sqrt{S_{\text{tot}}(1-\tilde{r}^2)}. \tag{26}$$

For fitting S_{hopf}, our baseline initial parameters $(\tilde{\sigma}, \tilde{\mu}, \tilde{\omega}_0)$ are set to satisfy

$$S_{\max} = \frac{\tilde{\sigma}^2}{4\pi\tilde{\mu}^2}\left(1 + \frac{\tilde{\mu}^2}{\tilde{\mu}^2 + 4\tilde{\omega}_0^2}\right), \qquad S_{\text{tot}} = -\frac{\tilde{\sigma}^2}{2\tilde{\mu}}, \qquad \omega_{\text{dom}} = \tilde{\omega}_0 \tag{27}$$

which are the analytical expressions for the peak in S_{hopf}, its total variation, and dominant frequency respectively. This gives an initialisation of

$$\tilde{\sigma} = \sqrt{-2\tilde{\mu}S_{\text{tot}}}, \quad \tilde{\mu} = -\frac{1}{3\pi S_{\max}}\left(S_{\text{tot}} + \alpha^{\frac{1}{3}} + \frac{S_{\text{tot}}^2 - 12\pi^2\omega_{\text{dom}}^2 S_{\max}^2}{\alpha^{\frac{1}{3}}}\right), \quad \tilde{\omega}_0 = \omega_{\text{dom}} \tag{28}$$

where

$$\begin{aligned}\alpha = {} & S_{\text{tot}}^3 + 9\pi^2\omega_{\text{dom}}^2 S_{\max}^2 S_{\text{tot}} \\ & + 3\sqrt{3}\pi\sqrt{64\pi^4\omega_{\text{dom}}^6 S_{\max}^6 - 13\pi^2\omega_{\text{dom}}^4 S_{\max}^4 S_{\text{tot}}^2 + 2\omega_{\text{dom}}^2 S_{\max}^2 S_{\text{tot}}^4}\end{aligned} \tag{29}$$

Finally, for fitting S_{null}, the baseline initial parameter $\tilde{\sigma}$ satisfies

$$S_{\text{tot}} = \tilde{\sigma}^2 \tag{30}$$

which is the analytical expression for the total variation of S_{null} (over the frequency domain $[-\pi, \pi]$). This simply gives us the baseline initialisation parameter of $\tilde{\sigma} = \sqrt{S_{\text{tot}}}$.

Once the baseline initialisation parameters are found, we can run the optimisation procedure to find the best fit. In cases where the power spectrum is non-typical, doing a sweep over a set of initialisation parameters increases the likelihood of finding the best fit. So we provide the option for the algorithm to sweep over $[0.5, 1, 1.5]$ times the baseline initialisation value for each parameter, and choose the best fit (lowest AIC score) Doing the sweep entails 3^3 initialisation settings for S_{hopf}, 3^2 settings for S_{fold} and S_{flip}, and 3 settings for S_{null}. This is a total of 48 initialisation settings, as opposed to 4, where no sweep is performed, so is more computationally expensive, but more likely to find a global minimum.

2.2 Analytical approximations of EWS

Here, we derive analytical approximations for early warning signals (EWS) using normal form bifurcations,[9] and standard tools from stochastic processes.[10,11] In particular, we consider EWS that precede each type of local bifurcation of codimension-one in continuous and discrete-time systems. The consideration of global bifurcations is beyond the scope of this work (and would require non-local methodologies). Each local bifurcations is accompanied by a particular (although not unique) movement of the eigenvalues of the Jacobian matrix. This eigenvalue movement results in changes to the statistical properties of fluctuations in the system state, which can serve as EWS. There are two generically different ways a local bifurcation can be induced in a continuous-time system, and three ways in a discrete-time system, as illustrated in Supplementary Figure 1. For each case, we derive the corresponding EWS, that can be used to preempt bifurcations that fall into this category. Table Table 1 summarises these derivations.

A glossary describing the relevant mathematical terms is provided at the end of this document for convenience. Glossary terms in the text are linked and in colour (online).

2.2.1 Continuous-time systems

Consider the generic continuous-time dynamical system depending on a single parameter

$$\dot{x} = f(x, \alpha), \quad x \in \mathbb{R}^n, \quad \alpha \in \mathbb{R}^1, \tag{31}$$

where f is a smooth function governing the deterministic dynamics of the system. We assume that the system sits in some stable equilibrium x_0, and therefore all eigenvalues of the Jacobian matrix about x_0 have negative real part. Upon varying α, a local bifurcation occurs at the point where an eigenvalue attains positive real part. There are generically only two possible ways this can occur.[9] Either a real eigenvalue becomes positive, or a complex conjugate pair attain positive real part (Figure 1). Both yield different bifurcations, and importantly, different EWS.

Case 1: A single real eigenvalue becomes positive. In the case where a real eigenvalue becomes positive, the system undergoes either a Fold, Transcritical or Pitchfork bifurcation, depending on higher order terms that are not captured by the eigenvalues. This unfortunately means that EWS using solely local stability properties of the system cannot distinguish these bifurcations in advance.

To derive generic EWS for these bifurcations, it is appropriate to consider them in their normal form, to which all other bifurcations of the same type are locally topologically equivalent.[9] Preceding each bifurcation, the linearised dynamics about the stable state of the system is identical, and given by

$$\frac{dx}{dt} = \lambda x, \tag{32}$$

where x represents the displacement from the equilibrium, and λ is the eigenvalue governing the stability of the system ($\lambda < 0$). Assuming the system is subject to small additive white noise, it obeys

$$\frac{dx}{dt} = \lambda x + \sigma \xi(t) \tag{33}$$

where $\xi(t)$ is a Gaussian white noise process and σ is the noise amplitude. We can now compute statistics of this process such as variance and autocorrelation, which can serve as EWS. We also note that this process is an Ornstein-Uhlenbeck process (OUp), whose properties are well-studied.[10] Integrating the expression gives

$$x(t) = x(0)e^{\lambda t} + \sigma e^{\lambda t} \int_0^t \xi(t') e^{-\lambda t'} dt', \tag{34}$$

where $x(0)$ is the initial position of the process. We are interested in the statistics of the process at stationarity, and so may safely drop the transient term, to arrive at

$$x(t) = \sigma e^{\lambda t} \int_0^t \xi(t') e^{-\lambda t'} dt'. \tag{35}$$

Note that in the limit as $\lambda \to 0$, the restoring forces vanish resulting in a random walk

$$x(t) = \sigma \int_0^t \xi(t') dt'. \tag{36}$$

We now compute the autocovariance function, from which many EWS can be derived. The autocovariance function of the stochastic process $x(t)$ is defined as

$$\phi(\tau) = \langle x(t)x(t+\tau) \rangle \tag{37}$$

where τ is a lag time, and we use $\langle ... \rangle$ to denote the expectation of a random variable. Inserting Eqn (33) into this expression yields

$$\phi(\tau) = \sigma^2 e^{2\lambda t + \lambda \tau} \int_0^t dt' \int_0^{t+\tau} dt'' \langle \xi(t')\xi(t'') \rangle e^{-\lambda(t'+t'')}. \tag{38}$$

By the assumption of white noise being uncorrelated, it satisfies[10]

$$\langle \xi(t')\xi(t'') \rangle = \delta(t' - t'') \tag{39}$$

where δ is the Dirac Delta function. The autocovariance may then be simplified and integrated to get

$$\phi(\tau) = \frac{-\sigma^2}{2\lambda} e^{\lambda \tau} (1 - e^{2\lambda t}) \tag{40}$$

which, in the stationary regime ($t \to \infty$) is

$$\phi(\tau) = \frac{-\sigma^2}{2\lambda} e^{\lambda \tau}. \tag{41}$$

This yields the familiar expressions for the variance and autocorrelation of the OUp, given by

$$\boxed{\mathrm{Var}(x) = \phi(0) = \frac{-\sigma^2}{2\lambda}} \tag{42}$$

and

$$\boxed{\rho(\tau) = \frac{\phi(\tau)}{\phi(0)} = e^{\lambda \tau}} \tag{43}$$

where τ is the lag-time. Note that as any one of these bifurcations is approached, λ approaches zero from below, causing variance and autocorrelation to increase monotonically to infinity and one respectively.

The power spectrum of the process can be computed using the Wiener-Khinchin Theorem, which states that for a stationary stochastic process, the power spectrum is the Fourier Transform of the autocovariance function.[11] We use the convention

$$S(\omega) = \frac{1}{2\pi} \int_{-\infty}^{\infty} e^{i\omega\tau} \phi(\tau) d\tau, \tag{44}$$

which for a real stochastic process (and given $\phi(\tau) = \phi(-\tau)$) may be reduced to

$$S(\omega) = \frac{1}{\pi} \int_0^{\infty} \cos(\omega\tau)\phi(\tau) d\tau. \tag{45}$$

Inserting the autocorrelation from Eqn. (41) we get

$$S(\omega) = -\frac{\sigma^2}{2\lambda} \frac{1}{\pi} \int_0^{\infty} \cos(\omega\tau) e^{\lambda\tau} d\tau \tag{46}$$

which integrates to give

$$\boxed{S(\omega) = \frac{\sigma^2}{2\pi} \frac{1}{\omega^2 + \lambda^2}.} \tag{47}$$

Case 2: A complex-conjugate pair of eigenvalues attain positive real part. In the case where a complex-conjugate pair of eigenvalues attain positive real part, the system either undergoes a super-critical (smooth) or a sub-critical (discontinuous) Hopf bifurcation, again, depending on higher terms that are not captured by the eigenvalues. Preceding each bifurcation in their normal form, the linearised dynamics about the stable state are given by

$$\frac{dx}{dt} = \mu x - \omega_0 y \tag{48}$$

$$\frac{dy}{dt} = \omega_0 x + \mu y, \tag{49}$$

where x and y represent displacements from the equilibrium in perpendicular directions, and the eigenvalues governing the stability of the system are now given by $\lambda = \mu \pm i\omega_0$. Note that a system must have at least two dimensions to undergo a Hopf bifurcation. To investigate EWS preceding these bifurcations, we include additive white noise to each of these components, to get the system

$$\frac{d\vec{x}}{dt} = J\vec{x} + B\vec{\xi}(t), \tag{50}$$

where $\vec{\xi}$ is a vector of Gaussian white noise processes (ξ_1, ξ_2), B is a matrix of noise amplitudes $\mathrm{diag}(\sigma_1, \sigma_2)$ and J is the Jacobian matrix given by

$$J = \begin{pmatrix} \mu & -\omega_0 \\ \omega_0 & \mu \end{pmatrix}. \tag{51}$$

The stochastic process in (50) is now a multi-variate OUp which, although more involved, can be subject to a similar treatment as the one-dimensional case. Integrating the process gives

$$\vec{x}(t) = e^{Jt}\vec{x}(0) + e^{Jt} \int_0^t e^{-Jt'} B\vec{\xi}(t') dt', \tag{52}$$

which has stationary dynamics given by

$$\vec{x}(t) = e^{Jt} \int_0^t e^{-Jt'} B\vec{\xi}(t') dt'. \tag{53}$$

The covariance matrix of a multi-dimensional process is defined as

$$\Sigma = \langle \vec{x}(t)\vec{x}^T(t) \rangle \tag{54}$$

which in this case gives

$$\Sigma = \int_0^t e^{J(t-t')} BB^T e^{J^T(t-t')} dt'. \tag{55}$$

In this derivation we used the property of independent white noise sources, $\langle\vec{\xi}(t)\vec{\xi}^T(t')\rangle = I\delta(t-t')$. Since the stochastic process is stationary, we have

$$\frac{d\Sigma}{dt} = 0, \tag{56}$$

and so taking the time derivative of (55) gives

$$J\Sigma + \Sigma J^T + BB^T = 0, \tag{57}$$

which is known as the continuous Lyapunov equation. In two dimensions, a closed form solution is known[10] and given by

$$\Sigma = \frac{-(J-(\mathrm{Tr}\,J)I)BB^T(J-(\mathrm{Tr}\,J)I)^T - \mathrm{Det}\,J BB^T}{2\,\mathrm{Tr}\,J\,\mathrm{Det}\,J}, \tag{58}$$

which we can use to find the variance in system variables preceding a Hopf bifurcation. Using the Jacobian matrix in (51), the variance of each variable is given by

$$\mathrm{Var}(x) = \Sigma_{11} = \frac{\sigma_1^2}{2(-\mu)} + \frac{\omega_0^2(\sigma_2^2-\sigma_1^2)}{4(-\mu)(\mu^2+\omega_0^2)}, \tag{59}$$

$$\mathrm{Var}(y) = \Sigma_{22} = \frac{\sigma_2^2}{2(-\mu)} + \frac{\omega_0^2(\sigma_1^2-\sigma_2^2)}{4(-\mu)(\mu^2+\omega_0^2)}, \tag{60}$$

and the covariance term satisfies

$$\mathrm{Cov}(x,y) = \Sigma_{12} = \frac{\omega_0(\sigma_2^2-\sigma_1^2)}{4(\mu^2+\omega_0^2)}. \tag{61}$$

The autocovariance matrix is defined as

$$\phi(\tau) = \langle\vec{x}(t+\tau)\vec{x}^T(t)\rangle. \tag{62}$$

One can readily show using Eqn (50) and the statistical properties of white noise, that

$$\frac{\partial\phi}{\partial\tau} = J\phi, \tag{63}$$

which can be solved to give

$$\phi(\tau) = e^{J\tau}\Sigma \tag{64}$$

where $\Sigma = \phi(0)$. So one can directly compute the autocovariance matrix ϕ from the covariance matrix Σ. For the Jacobian in (51), this reduces to

$$\phi(\tau) = e^{\mu\tau}\begin{pmatrix}\cos(\omega_0\tau) & -\sin(\omega_0\tau)\\ \sin(\omega_0\tau) & \cos(\omega_0\tau)\end{pmatrix}\Sigma. \tag{65}$$

The power spectrum of each variable can be computed as before using the Wiener Khinchin Theorem. Since these expressions are long, we work with the leading order terms below under two different assumptions. (1) The noise amplitudes for each variable are similar, and (2) the system is very close to the bifurcation point, allowing us to assume that μ is small.

To leading order in noise amplitude difference

Here, we assume that the noise amplitudes σ_1 and σ_2 are similar, so $\sigma_2 = \sigma_1 + \epsilon$ where ϵ is small. Then to leading order, we have

$$\boxed{\mathrm{Var}(x) = -\frac{\sigma_1^2}{2\mu} + O(\epsilon),} \tag{66}$$

similar to the case of a single real eigenvalue. Note that all statistical expressions for the variable y can be obtained from those of x by interchanging σ_1 and σ_2. The autocorrelation coefficient $\rho_{xx} = \frac{\phi_{xx}(\tau)}{\phi_{xx}(0)}$ to leading order is

$$\boxed{\rho_{xx}(\tau) = e^{\mu\tau}\cos(\omega_0\tau) + O(\epsilon).} \tag{67}$$

From this expression, the power spectrum is computed by taking the Fourier transform,

$$S_{xx}(\omega) = -\frac{\sigma_1^2}{2\mu}\frac{1}{\pi}\int_0^\infty e^{\mu\tau}\cos(\omega_0\tau)\cos(\omega\tau)d\tau + O(\epsilon), \tag{68}$$

which can be evaluated to give

$$\boxed{S_{xx}(\tau) = \frac{\sigma_1^2}{4\pi}\left(\frac{1}{(\omega+\omega_0)^2+\mu^2} + \frac{1}{(\omega-\omega_0)^2+\mu^2}\right) + O(\epsilon).} \tag{69}$$

To leading order in proximity to bifurcation

To consider the case with dissimilar noise amplitudes, we can instead simplify matters by considering small μ, which assumes the system is close to the Hopf bifurcation. Taylor expanding about $\mu = 0$ gives variance

$$\mathrm{Var}(x) = \frac{\sigma_1^2+\sigma_2^2}{4(-\mu)} + O(\mu), \tag{70}$$

and autocorrelation

$$\rho_{xx}(\tau) = \cos(\omega\tau) + \mu\left(\tau\cos(\omega\tau) + \frac{\sigma_2^2-\sigma_1^2}{\sigma_1^2+\sigma_2^2}\frac{1}{\omega}\sin(\omega\tau)\right) + O(\mu^2). \tag{71}$$

Importantly, note that regardless of the noise amplitudes, the dominant term in the autocorrelation as the bifurcation is approached is $\cos(\omega\tau)$. This provides the distinguishing feature of a Hopf bifurcation, by taking positive or negative values depending on the lag time.

The $O(\mu)$ term in the power spectrum vanishes, and so can be computed to $O(\mu^2)$ as

$$S_{xx}(\omega) = \frac{1}{8\pi\omega_0}\left(\frac{2\sigma_1^2\omega_0 + (\sigma_1^2-\sigma_2^2)\omega}{\mu^2+(\omega+\omega_0)^2} + \frac{2\sigma_1^2\omega_0 + (\sigma_2^2-\sigma_1^2)\omega}{\mu^2+(\omega-\omega_0)^2}\right) + O(\mu^2), \tag{72}$$

which recovers recovers the 'noise-amplitude approximation' as $\sigma_1 \to \sigma_2$. We see that regardless of the noise amplitudes, the power spectrum is still maximised at $\omega = \pm\omega_0$ as the bifurcation is approached ($\mu \to 0$), providing the characteristic feature of an upcoming Hopf bifurcation.

2.2.2 Discrete-time systems

Case 1: A real eigenvalue moves above one In the case where a real eigenvalue moves above one, the system undergoes either a Fold, Transcritical or Pitchfork bifurcation, analogous to the case of a positive real eigenvalue in a continuous system. Preceding each of these bifurcations in their normal form, the linearised dynamics satisfy

$$x_{t+1} = \lambda x_t, \tag{73}$$

where x_t denotes the displacement from the equilibrium, and λ is the eigenvalue governing the stability of the system ($|\lambda| < 1$). Assuming small additive white noise, the discrete then obeys

$$x_{t+1} = \lambda x_t + \sigma \epsilon_t \tag{74}$$

where ϵ_t is a normal random variable, and σ is the noise amplitude. This equation represents an AR(1) process which is the discrete-time analogue of the OUp, and has been studied thoroughly in the statistics literature.[11]

The variance of this process can be computed by squaring both sides of (74) and taking the expectation, giving

$$\mathbb{E}[x_{t+1}^2] = \lambda^2 \mathbb{E}[x_t^2] + 2\lambda\sigma\mathbb{E}[\epsilon_t x_t] + \sigma^2 \mathbb{E}[\epsilon_t^2]. \tag{75}$$

Given that the process is stationary, $\mathbb{E}[x_{t+1}^2] = \mathbb{E}[x_t^2]$. The white noise term is a normal random variable, independent of x_t and with unit variance, hence $\mathbb{E}[\epsilon_t x_t] = 0$, and $\mathbb{E}[\epsilon_t^2] = 1$. The expression can then be simplified to obtain the variance,

$$\boxed{\mathrm{Var}(x) = \mathbb{E}[x_t^2] = \frac{\sigma^2}{1-\lambda^2},} \tag{76}$$

which diverges as the bifurcations are approached ($\lambda \to \pm 1$).

The lag-1 autocorrelation can be computed by multiplying (74) by x_t and taking expectations. This yields

$$\mathbb{E}[x_{t+1}x_t] = \lambda\mathbb{E}[x_t^2] + \sigma\mathbb{E}[\epsilon_t x_t] \tag{77}$$

which rearranges to

$$\rho_1 = \frac{\mathbb{E}[x_{t+1}x_t]}{\mathbb{E}[x_t^2]} = \lambda. \tag{78}$$

When applied recursively, one obtains the autocorrelation function

$$\boxed{\rho_\tau = \lambda^{|\tau|}.} \tag{79}$$

From this we see that as $\lambda \to 1$ (Fold/Transcritical/Pitchfork), the autocorrelation increases monotonically to 1 regardless of the lag-time. However, in the approach to the flip bifurcation, the autocorrelation increases or decreases depending on the lag time.

The power spectrum can be computed by taking the Discrete-Fourier-Transform of the autocovariance function

$$\phi_\tau = \mathbb{E}[x_t x_{t+\tau}] = \frac{\sigma^2\lambda^{|\tau|}}{1-\lambda^2} \tag{80}$$

which gives

$$S(\omega) = \frac{1}{2\pi} \sum_{\tau=-\infty}^{\infty} e^{-i\omega\tau} \phi_\tau \tag{81}$$

$$= \frac{1}{2\pi} \frac{\sigma^2}{1-\lambda^2} \sum_{\tau=-\infty}^{\infty} e^{-i\omega\tau} \lambda^{|\tau|}. \tag{82}$$

Noting that the infinite sum can be split into two geometric series, one obtains the power spectrum as

$$\boxed{S(\omega) = \frac{\sigma^2}{2\pi} \frac{1}{1+\lambda^2 - 2\lambda\cos(\omega)}.} \tag{83}$$

Plots of the power spectrum for $\lambda \to \pm 1$ are shown in Figure (). Note that

$$S(0) = \frac{1}{2\pi} \frac{1+\lambda}{1-\lambda} \tag{84}$$

which diverges as $\lambda \to 1$ as we expect for the zero-frequency component preceding these bifurcations. In contrast

$$S(\pi) = \frac{1}{2\pi} \frac{1-\lambda}{1+\lambda} \tag{85}$$

diverges when the Flip bifurcation is approached ($\lambda \to -1$), again as expected since the Flip bifurcation is the onset of oscillations of frequency π ($T = 2$).

Case 2: A real eigenvalue moves below minus one In the case where a real eigenvalue moves below minus one, the system undergoes something entirely different - a Flip bifurcation, that is not possible in the continuous system. The analytical approximations are the same as for the previous case, except the limiting behaviour preceding the bifurcation is obtained by taking $\lambda \to -1$.

Case 3: A complex-conjugate pair of eigenvalues attain a magnitude greater than one In this case, the system undergoes a Neimark-Sacker bifurcation,[9] which the discrete-time analogue of the Hopf bifurcation. Note this bifurcation is only possible in systems with dimension $n \geq 2$. The difference equation governing the linearised dynamics for this bifurcation is

$$x_{t+1} = r\cos\theta\, x_t - r\sin\theta\, y_t \tag{86}$$

$$y_{t+1} = r\sin\theta\, x_t + r\cos\theta\, y_t \tag{87}$$

where (x_t, y_t) is the (two-dimensional) displacement from equilibrium at time t, and $r \in (0, \infty)$ and $\theta \in (0, \pi)$ are parameters. With additive GWN, this system may be written

$$\vec{x}_{t+1} = J\vec{x}_t + B\vec{\epsilon}_t \tag{88}$$

where $\vec{\epsilon}_t$ is a vector of normal random variables (ϵ_1, ϵ_2), B is a matrix of noise amplitudes $\text{diag}(a_1, a_2)$ and J is the Jacobian matrix given by

$$J = \begin{pmatrix} r\cos\theta & -r\sin\theta \\ r\sin\theta & r\cos\theta \end{pmatrix}. \tag{89}$$

The eigenvalues of J are a complex-conjugate pair given by $\lambda = re^{\pm i\theta}$, corresponding to case (e) in Figure 1. When $r = 1$, the eigenvalues reach the unit circle and the Neimark-Sacker bifurcation bifurcation occurs. How do the EWS behave?

The stochastic difference equation in (88) is a VAR(1) process (vector autoregressive process of order one). The variance of each variable can be obtained from the covariance matrix, defined as

$$\begin{aligned}\Sigma &= \mathbb{E}(\vec{x}_{t+1}\vec{x}_{t+1}^T) &(90)\\ &= \mathbb{E}[(J\vec{x}_t + B\vec{\epsilon}_t^T)(\vec{x}_t^T J^T + \vec{\epsilon}_t^T B^T)] &(91)\\ &= J\Sigma J^T + BB^T &(92)\end{aligned}$$

where in the final step we have used properties of stationarity and independence of the noise. This is the discrete-time Lyapunov equation, which we can solve to find the covariance matrix. We solve this using symbolic computation in Mathematica. Expressions are long except for the case of equal noise amplitudes ($\sigma_1 = \sigma_2$), so we display those results here. However, differing noise amplitudes yields the same important features expected in the EWS. In the case of equal noise amplitude, the variance in the state variable x is given by

$$\boxed{\mathrm{Var}(x_t) = \frac{\sigma_1^2}{1-r^2},} \tag{93}$$

with the same scaling behaviour as in the one-dimensional case. We now compute the autocovariance matrix, which is defined as

$$\phi_\tau = \mathbb{E}[\vec{x}_{t+\tau}\vec{x}_t^T] \tag{94}$$

which can be shown to satisfy $\phi_{\tau+1} = J\phi_\tau$ using the definition and equation (88). Solving this difference equation gives

$$\begin{aligned}\phi_\tau &= J^\tau \phi_0 &(95)\\ &= J^\tau \Sigma, &(96)\end{aligned}$$

which, with the Jacobian in (89) becomes

$$\phi_\tau = r^\tau \begin{pmatrix} \cos(\theta\tau) & -\sin(\theta\tau) \\ \sin(\theta\tau) & \cos(\theta\tau) \end{pmatrix} \Sigma. \tag{97}$$

The autocorrelation for the variable x_t is then simply

$$\boxed{\rho_\tau = r^\tau \cos(\theta\tau),} \tag{98}$$

much like the autocorrelation preceding the Hopf bifurcation in the continuous-time case. We compute the power spectrum by taking the DFT of the autocovariance function for x_t

$$\phi_\tau = \frac{\sigma_1^2}{1-r^2} r^\tau \cos(\theta|\tau|) \tag{99}$$

where we have included the absolute value of τ in the exponent since the autocovariance function is symmetric in τ. The power spectrum is then

$$\begin{aligned}S(\omega) &= \frac{1}{2\pi}\sum_{\tau=-\infty}^{\infty} e^{-i\omega\tau}\phi_\tau &(100)\\ &= \frac{1}{2\pi}\frac{\sigma_1^2}{1-r^2}\sum_{\tau=-\infty}^{\infty} e^{-i\omega\tau} r^{|\tau|}\cos(\theta\tau). &(101)\end{aligned}$$

which can be written as a geometric series* and computed as

$$\boxed{S(\omega) = \frac{\sigma_1^2}{4\pi}\left(\frac{1}{1+r^2-2r\cos(\omega-\theta)} + \frac{1}{1+r^2-2r\cos(\omega+\theta)}\right).} \tag{102}$$

*use the relation $\cos(\theta\tau) = (e^{i\theta\tau} + e^{-i\theta\tau})/2$

2.3 Asymptotic properties of S_{max}

This section provides derivations of the asymptotic behaviour of S_{max} up to each local codimension-1 bifurcation. The derivations use the analytical approximations of the power spectrum, as derived earlier and displayed in Table 1. The asymptotic behaviour is derived in terms of the distance to the bifurcation d, which we define as the smallest difference between the current eigenvalue (of the Jacobian matrix that governs the local dynamics of the system) and a location at which a bifurcation occurs.

Continuous-time: Fold, Transcritical, Pitchfork. These bifurcations occur when a real eigenvalue attains positive real part, and so $d = |\lambda|$, where $\lambda \in \mathbb{R}^{\leq 0}$. The power spectrum for the normal form of these bifurcations is

$$S(\omega) = \frac{\sigma^2}{2\pi}\left(\frac{1}{\omega^2 + \lambda^2}\right) \tag{103}$$

where σ is the white noise amplitude. The maximum value of this function occurs at $\omega = 0$, and is

$$S_{\mathrm{max}} = \frac{\sigma^2}{2\pi d^2} \sim \frac{1}{d^2} \quad \text{as} \quad d \to 0. \tag{104}$$

Continuous-time: Hopf. The Hopf bifurcation occurs when a complex-conjugate pair of eigenvalues $\lambda_{\pm} = \mu \pm i\omega$ attain positive real part. The distance to the bifurcation in this case is $d = |\mu|$. The power spectrum for the normal form of this bifurcation is

$$S(\omega) = \frac{\sigma^2}{4\pi}\left(\frac{1}{(\omega - \omega_0)^2 + \mu^2} + \frac{1}{(\omega + \omega_0)^2 + \mu^2}\right). \tag{105}$$

This function attains maximum value at $\omega = \omega_0$, which may be written

$$S_{\mathrm{max}} = \frac{\sigma^2}{4\pi d^2}\left(1 + \frac{d^2}{4\omega_0^2 + d^2}\right). \tag{106}$$

As $d \to 0$, the bracketed term goes to 1 and we are left with the asymptotic behaviour

$$S_{\mathrm{max}} \sim \frac{1}{d^2} \quad \text{as} \quad d \to 0. \tag{107}$$

Discrete-time: Fold, Transcritical, Pitchfork. These bifurcations occur when a real eigenvalue reaches the value one from below, and so $d = 1 - \lambda$, where $\lambda \in \mathbb{R}$. The power spectrum for the normal forms of these bifurcations is

$$S(\omega) = \frac{\sigma^2}{2\pi}\left(\frac{1}{1 + \lambda^2 - 2\lambda\cos(\omega)}\right), \quad \omega \in [-\pi, \pi]. \tag{108}$$

Provided that $\lambda > 0$, which is the region in which these bifurcations occur, the maximum value of this function occurs at $\omega = 0$ and is

$$S_{\mathrm{max}} = \frac{\sigma^2}{2\pi(1-\lambda)^2} = \frac{\sigma^2}{2\pi d^2} \sim \frac{1}{d^2} \quad \text{as} \quad d \to 0. \tag{109}$$

Discrete-time: Flip (period-doubling). This bifurcation occurs when a real eigenvalue reaches the value negative one from above, and so $d = \lambda + 1$. The power spectrum for the normal form of the Flip bifurcation is the same as for the previous bifurcations, however. The maximum of this power spectrum in the region $\lambda < 0$ is

$$S_{\mathrm{max}} = \frac{\sigma^2}{2\pi(1+\lambda)^2} = \frac{\sigma^2}{2\pi d^2} \sim \frac{1}{d^2} \quad \text{as} \quad d \to 0, \tag{110}$$

Discrete-time: Neimark-Sacker. The Neimark-Sacker bifurcation occurs when a complex conjugate pair of eigenvalues $\lambda = re^{\pm\omega_0}$ obtain unit magnitude, and so $d = 1 - r$. The power spectrum for the normal form Neimark-Sacker bifurcation is

$$S(\omega) = \frac{\sigma^2}{4\pi}\left(\frac{1}{1 + r^2 - 2r\cos(\omega - \omega_0)} + \frac{1}{1 + r^2 - 2r\cos(\omega + \omega_0)}\right). \tag{111}$$

This takes a maximum value at $\omega = \omega_0$ may be written

$$S_{\max} = \frac{\sigma^2}{4\pi}\left(\frac{1}{(1-r)^2} + \frac{1}{1 + r^2 - 2r\cos(2\omega_0)}\right) \tag{112}$$

$$= \frac{\sigma^2}{4\pi d^2}\left(1 + \frac{d^2}{d^2 + 2(1-d)(1-\cos(2\omega_0))}\right) \tag{113}$$

$$\sim \frac{1}{d^2} \quad \text{as} \quad d \to 0, \tag{114}$$

since the bracketed term goes to 1.

3 Supplementary Note

3.1 Local bifurcations in higher-dimensional systems

Here, we show how results from random matrix theory suggest that higher-dimensional systems are more prone to oscillatory bifurcations than their simplified models would suggest. Given how autocorrelation behaves in non-trivial ways prior to these bifurcations (Figure 1), this work provides further motivation for the development of a metric that is robust to the type of local bifurcation (S_{max}).

This section is organised as follows. We begin by outlining how random matrix theory has been used in the past to draw connections between stability and complexity in complex systems, particularly ecological networks. We then make hypotheses about the types of local bifurcations that are likely to occur, given the type of interactions between the individual components of the system. Finally, we run simulations of random networks to test these hypotheses.

May's complexity-stability criterion

It was over 40 years ago, when May established a relationship between complexity and stability in food webs with a large number of species,[12] using results from random matrix theory.[13,14] Subsequently, random matrix theory has been used extensively to draw connections between complexity and stability in ecological networks.[15,16] In this context, complexity refers to a quantity weighted by connectance C (the probability that any pair of species interact), the average interaction strength between the species α, and the total number of species in the system n. May's result states that for large n, the system will almost certainly be stable provided

$$\alpha\sqrt{nC} < d, \tag{115}$$

where d is a coefficient that corresponds to the strength of self-regulatory effects (the stability of each species in isolation from the others). The system is almost certainly unstable if this condition does not hold. As a consequence, systems are likely to be unstable if they possess a large number of variables, are highly connected, or have weak self-regulatory effects. This result is in accordance with the empirical observation that species-rich ecosystems tend to have lower connectance than those with a smaller number of species.[17]

The mathematics underlying this stability criterion comes from the field of random matrix theory, which has established remarkable theorems concerning the spectrum of eigenvalues for large, random matrices.[13,18,19] A random matrix is simply a matrix whose coefficients are drawn from a specific set probability distributions. Originally formalised in physics for studying statistical models of heavy nuclei,[13] the field has grown rapidly, finding applications in e.g. data analysis, number theory and optimal control. Though how can random matrix theory bring insights to ecology?

An ecosystem can be modelled as a network, where each node corresponds to the dynamics of a single species, and connections capture the species interactions, which are in general non-linear. At equilibrium, the stability of the system may be obtained from the Jacobian matrix, whose elements correspond to the type and strength of interaction between each pair of species. Without loss of generality, we may assume that each of these elements has been drawn from some probability distribution. Random matrix theory can tell us the expected distribution of eigenvalues, given a probability distribution from which the matrix elements are drawn. This is useful, since these eigenvalues determine stability of the system, and to an extent, characterise possible bifurcations nearby.

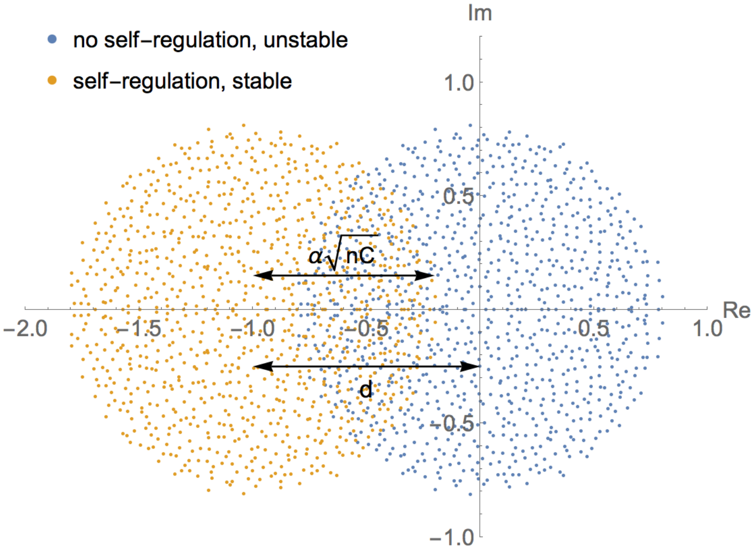

Supplementary Figure 11. Illustration of May's stability criterion. Shown are the eigenvalue spectra of two 1000×1000 matrices whose elements are, with probability C, drawn from a normal distribution with mean 0 and variance α^2 and otherwise set to 0. Without the assumption of self-regulating terms (blue) the corresponding system is unstable due to positive eigenvalues. With self-regulating terms (incorporated by replacing the diagonal elements of the matrix by $-d$), the corresponding system is stable since all eigenvalues are negative (orange). The radius of the spectrum is $\alpha\sqrt{nC}$ which must be less than d for the system to be stable (with high probability). This is the stability criterion that May derived. Parameters $d = -1$, $\alpha = 0.025$, $n = 1000$, $C = 1$.

The Circular Law

The result from random matrix theory that produced May's stability criterion (115) is Wigner's 'semicircle law',[13] which has now been proven in greater generality, and called the 'circular law'.[19] The circular law is stated simply as follows. Assuming each element of the an $n \times n$ matrix A is drawn from a probability distribution with mean zero and variance α^2, then the spectrum of eigenvalues of A converges to the uniform distribution over the disk with radius $\alpha\sqrt{n}$ centred at the origin. This property has become known as 'universality'.[19] Since ecosystems are typically sparsely connected, we can add to this and assume that each element of the matrix is assigned zero with probability $1 - C$ and is drawn from the given distribution with probability C (defining C as the connectance). The variance of the resulting distribution is reduced to $C\sigma^2$ and so using the property of universality, the eigenvalues of A converge to a disk of radius $\alpha\sqrt{nC}$ centred at the origin (Figure 11, blue). Moreover, if one assumes that in isolation, each species exhibits stable dynamics (incorporated by setting the diagonal elements of A to $-d$), then the eigenvalue spectrum is is shifted to the left by d units (Figure 11, orange). It is now clear that for all eigenvalues in this system to be less than zero (with high probability) we require that the radius of the circle $\alpha\sqrt{nC}$ is less than d, which is the complexity-stability relationship that May put forward (115).

Interaction types and stability

Note that in deriving the stability criterion in Eq. 115, May considered large ecological networks in which all interactions (a_{ij}) are drawn from a single probability distribution, and thus each pair of species interacts with the same probability, and with a completely random type of interaction. Allesina and Tang[15] built upon this work by considering networks where the proportion of interaction types (e.g. mutualistic, cooperative, exploitative) could be specified. For example, imposing a predator prey interaction would necessitate inter-

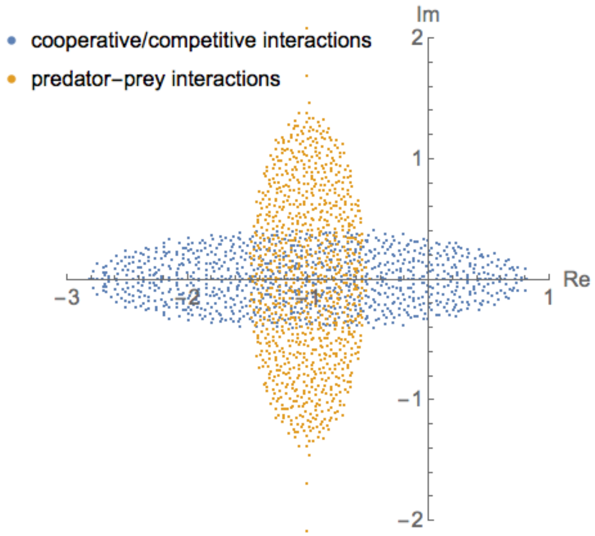

Supplementary Figure 12. Eigenvalue spectrum for matrices with imposed interaction types. Reconstruction of work by Allesina and Tang.[15] Shown are the eigenvalue spectra for two simulated 1000×1000 matrices. Blue dots correspond to eigenvalues of a matrix generated with half cooperative interactions and half competitive interactions. Orange dots correspond to eigenvalues of a matrix simulated solely with predator-prey interactions. This shows how predator-prey interactions are stabilising, whereas cooperative / competitive interactions are destabilising. See Allesina and Tang[15] for simulation methods and derived forms for the semi-major axis of each ellipse, which gives the stability threshold. Parameters $d = -1$, $\alpha = 0.05$, $n = 1000$, $C = 0.5$.

action terms a_{ij} and a_{ji} having opposite signs, imposing a mutualistic interaction would necessitate a_{ij} and a_{ji} both being positive. We introduce their work here, since we find that the proportion of each interaction type in the system not only has a significant effect on stability, but also on the way in which stability may be lost, i.e. the type of bifurcation.

Allesina and Tang found that imposing interaction types on the system morphed the eigenvalue spectrum into an ellipse (Figure 12). In particular, exploitative interactions (such as those found in predator-prey systems) narrowed the ellipse, reducing the size of the largest eigenvalue and therefore enhancing stability. Cooperative and competitive interactions widened the ellipse, increasing the size of the largest eigenvalue and therefore diminishing stability. This profound result demonstrates that random matrix theory has a lot to offer in understanding what makes complex systems stable. We suggest that random matrix theory can also give insight into the way in which a complex system destabilises, i.e. the type of bifurcation that it go through.

Random matrix theory and bifurcation type

Although much progress has been made on the stability of high-dimensional systems in theoretical and applied work,[15,16,20,21] the description of ensuing dynamics following an instability is less clear. The loss in stability of a resident stable state can lead to all sorts of behaviour, including e.g. oscillations, chaos, or a transition to some faraway attractor. The immediate dynamics following this instability may be understood via the underlying bifurcation. One can characterise the bifurcation to a certain extent by the behaviour of the destabilising eigenvalues (Figure 1).

We will consider continuous-time systems here. Therefore the two possible cases that result in the system destabilising are a real eigenvalue becoming positive (Fold/Transcritical/Pitchfork) or a complex-conjugate

pair obtaining positive real part (Hopf). We investigate the extent to which these large random matrices resemble Hopf bifurcations when they are perturbed to a point of instability.

Notice that a significant portion of the eigenvalues in these spectra have a non-zero imaginary part (they lie off the real axis). In fact, there is a remarkable inverse power law relationship between the dimension of a random matrix and the expected proportion of real eigenvalues that it possesses[18] (Figure 13). As a consequence, the proportion of real eigenvalues drops off rapidly as the system size increases. Moreover, the type of interactions in the system has a significant effect, with predator prey interactions resulting in a smaller proportion of real eigenvalues, compared to system made up of cooperative and competitive interactions. Due to these high proportions of complex eigenvalues, we hypothesise that high-dimensional systems, particularly those with predator-prey interactions, are more prone to Hopf bifurcations than systems with a small number of interacting components.

To test this hypothesis, we numerically simulate large random matrices with various proportions of interaction types using the methods of Allesina and Tang.[15] We then gradually reduce the self-regulating strength of a random selection of the species to force the system to destabilise. At the point where the system destabilises, we record whether this was due to a real eigenvalue or a complex conjugate pair. We do this for 1000 simulated community matrices and record the proportion of instabilities that correspond to a Hopf bifurcation. This is then done over a variety of system sizes and interaction types. Results are shown in Figure 14. We find that the likelihood of the system encountering a Hopf bifurcation increases with species diversity (defined as the number of interacting species in the system) as expected. One might have expected a higher proportion in general for the high-dimensional systems due to the minute proportion of real eigenvalues. However, since the eigenvalue spectrum tends to an ellipse for large matrix dimensions, the eigenvalues with the largest real part will tend to lie on the real axis, and so with a reasonable probability will be the first eigenvalues to cause destabilisation. The simulations also demonstrate that predator-prey interactions promote Hopf bifurcations, which is in line with the observed oscillatory nature of predator-prey systems. Systems dominated by cooperative or competitive interactions are less conducive to oscillations.

In summary, this investigation has shown that Hopf bifurcations are an abundant feature of high-dimensional systems, particularly those with exploitative interactions between agents. Given that Hopf bifurcations are also candidates for critical slowing down, a metric that is able to distinguish them from other local bifurcations should be a useful addition to the current ecosystem forecasting tools.

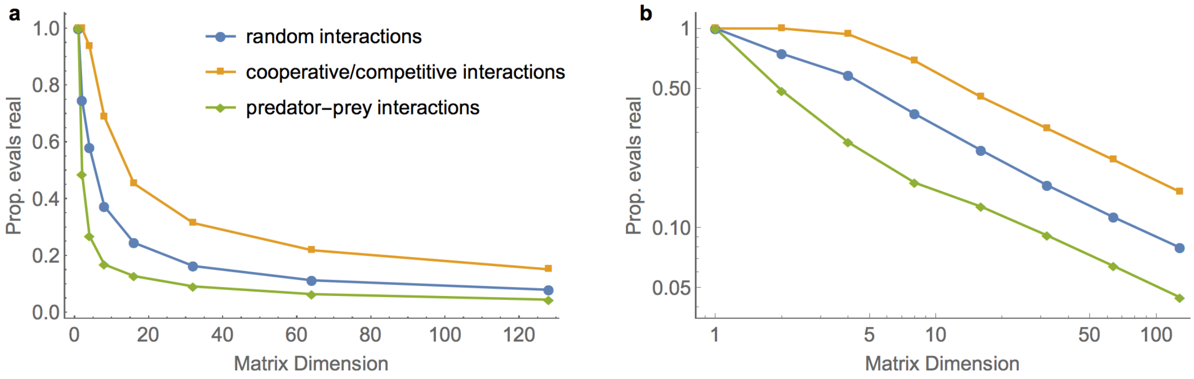

Supplementary Figure 13. Proportion of real eigenvalues rapidly decreases with system size. **a.** For select matrix dimensions, we simulate an ensemble of 1000 matrices for each of the interaction regimes. Points show the proportion of real eigenvalues amongst the entire ensemble. This proportion drops off much faster in predator-prey systems than for systems dominated by competitive and cooperative interactions. **b.** The same data on a log-log plot, demonstrating approximate power law behaviour for large dimensions. The power law exponent for the case of random interactions is approximately $\alpha = -0.54$, in agreement with early work by Sommers et al.[18] Parameters $d = -1$, $\alpha = 0.05$, $C = 0.5$.

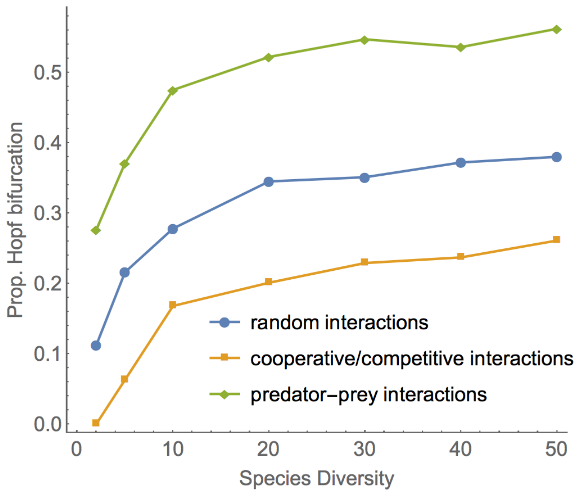

Supplementary Figure 14. Species diversity influences likelihood of Hopf bifurcation. For specific species diversity and interaction types, we simulated 1000 community matrices and incrementally perturbed them until they destabilised. The plot shows the proportion of these instabilities that manifested as Hopf bifurcations, showing the increasing trend with species diversity, and favouring predator-prey interactions among others.

Glossary

AR(1) process Autoregressive model of order one. A stochastic difference equation describing a mean-reverting random walk. Discrete-time analogue of the continuous-time Ornstein-Uhlenbeck process. 21, 31

Bifurcation A topological change in the behaviour a dynamical system as a parameter varies smoothly past a point. 16

Codimension The number of independent conditions (parameters) determining a bifurcation. 16

Eigenvalues Quantify the local stability of a system in equilibrium. The system is stable so long as all eigenvalues are negative (magnitude less than one) in continuous (discrete)-time systems. 16

Flip/Period-doubling bifurcation A local bifurcation involving a transition to behaviour twice the period of previous behaviour. Occurs in discrete-time systems. Characterised by a real eigenvalue passing through negative one.. 22

Fold/Saddle-node bifurcation A local bifurcation involving collision of a stable and unstable fixed point, resulting in a discontinuous transition to an alternate stable state. Characterised by a real eigenvalue passing through zero (one) in continuous (discrete)-time systems. Often associated with a 'tipping point'. 16

Gaussian white noise A random signal consisting of Gaussian distributed points with zero temporal correlation. 16, 18

Global bifurcation Induced by changes in global system properties such as the collision of basin boundaries. Not detectable from local stability analysis.. 16

Hopf bifurcation A local bifurcation involving a transition from a stable equilibrium to stable oscillations. Occurs in continuous-time systems. Characterised by a complex-conjugate pair of eigenvalues attaining positive real part. 18, 22

Jacobian matrix Describes the local (linear) dynamics of a system about an equilibrium state. Also known as the 'Community matrix' in ecology. 16

Local bifurcation Induced by changes in local stability (eigenvalues of the Jacobian matrix). Detectable from local stability analysis.. 16

Neimark-Sacker bifurcation A local bifurcation involving a transition from a stable equilibrium to stable oscillations. Occurs in discrete-time systems (analogous to the Hopf). Characterised by a complex-conjugate pair of eigenvalues attaining magnitude greater than one. 22

Normal form The simplest form of a bifurcation to which all others of the same type are locally topologically equivalent.. 16, 18, 21

Ornstein-Uhlenbeck process A stochastic differential equation describing a mean-reverting random walk. Continuous-time analogue of the discrete-time AR(1) process. 16, 31, 32

Pitchfork bifurcation A local bifurcation involving a transition between one and three equilibria. Characterised by a real eigenvalue passing through zero (one) in continuous (discrete)-time systems. Occurs in systems with symmetry. 16

Stochastic process A process that generates a random variable through time, that can be characterised by the mean, variance, and higher statistical moments. They are widely used as mathematical models for systems that involve 'randomness'.. 16

Transcritical bifurcation A local bifurcation involving a smooth transition between colliding stable states. Characterised by a real eigenvalue passing through zero (one) in continuous (discrete)-time systems. 16

VAR(1) process Vector autoregressive model of order one. A stochastic difference equation describing a mean-reverting random walk in multiple dimensions. Discrete-time analogue of the multi-variate Ornstein-Uhlenbeck process. 23

References

1. Fussmann, G. F., Ellner, S. P., Shertzer, K. W. & Hairston Jr, N. G. Crossing the Hopf bifurcation in a live predator-prey system. *Science* **290,** 1358–1360 (2000).
2. Politis, D. N. & Romano, J. P. The stationary bootstrap. *Journal of the American Statistical association* **89,** 1303–1313 (1994).
3. Welch, P. The use of fast Fourier transform for the estimation of power spectra: a method based on time averaging over short, modified periodograms. *IEEE Transactions on audio and electroacoustics* **15,** 70–73 (1967).
4. Dakos, V. *et al.* Methods for detecting early warnings of critical transitions in time series illustrated using simulated ecological data. *PloS one* **7,** e41010 (2012).
5. Beninca, E., Dakos, V., Van Nes, E. H., Huisman, J. & Scheffer, M. Resonance of plankton communities with temperature fluctuations. *The American Naturalist* **178,** E85–E95 (2011).
6. Akaike, H. in *Selected Papers of Hirotugu Akaike* 371–386 (Springer, 1987).
7. Newville, M. *et al.* LMFIT: non-linear least-square minimization and curve-fitting for Python. *Astrophysics Source Code Library* (2016).
8. Wagenmakers, E.-J. & Farrell, S. AIC model selection using Akaike weights. *Psychonomic bulletin & review* **11,** 192–196 (2004).
9. Kuznetsov, Y. A. *Elements of applied bifurcation theory* (Springer Science & Business Media, 2013).
10. Gardiner, C. W. *et al. Handbook of stochastic methods* (Springer Berlin, 1985).
11. Box, G. E., Jenkins, G. M., Reinsel, G. C. & Ljung, G. M. *Time series analysis: forecasting and control* (John Wiley & Sons, 2015).
12. May, R. M. Will a large complex system be stable? *Nature* **238,** 413–414 (1972).
13. Wigner, E. P. On the distribution of the roots of certain symmetric matrices. *Annals of Mathematics,* 325–327 (1958).
14. Mehta, M. L. *Random matrices* (Elsevier, 2004).
15. Allesina, S. & Tang, S. Stability criteria for complex ecosystems. *Nature* **483,** 205–208 (2012).
16. Mougi, A. & Kondoh, M. Diversity of interaction types and ecological community stability. *Science* **337,** 349–351 (2012).
17. Montoya, J. M. & Solé, R. V. Topological properties of food webs: from real data to community assembly models. *Oikos* **102,** 614–622 (2003).
18. Sommers, H., Crisanti, A., Sompolinsky, H. & Stein, Y. Spectrum of large random asymmetric matrices. *Physical review letters* **60,** 1895 (1988).
19. Tao, T. & Vu, V. Random matrices: the circular law. *Communications in Contemporary Mathematics* **10,** 261–307 (2008).
20. Coyte, K. Z., Schluter, J. & Foster, K. R. The ecology of the microbiome: networks, competition, and stability. *Science* **350,** 663–666 (2015).
21. Kuehn, C., Zschaler, G. & Gross, T. Early warning signs for saddle-escape transitions in complex networks. *Scientific reports* **5** (2015).
